# Biportal endoscopic foraminotomy of the L7–S1 neuroforamen in dogs: Description of surgical technique and ex vivo comparison with conventional open dorsolateral foraminotomy

**DOI:** 10.1111/vsu.70096

**Published:** 2026-03-12

**Authors:** Dimitrios Bekiaridis, Antonio Pozzi, Frank Steffen, Julian Guevar, Lucas A. Smolders

**Affiliations:** ^1^ Clinic for Small Animal Surgery, Vetsuisse‐Faculty University of Zurich Zurich Switzerland; ^2^ Clinic for Small Animal Neurology, Vetsuisse‐Faculty University of Zurich Zurich Switzerland; ^3^ Clinic for Small Animal Neurology, Vetsuisse‐Faculty University of Bern Bern Switzerland; ^4^ Bessy's Small Animal Clinic, IVC Evidensia Watt Switzerland

## Abstract

**Objective:**

(1) To establish a surgical technique for minimally invasive biportal endoscopic foraminotomy (BEF) of L7–S1 in dogs using arthroscopic equipment. (2) To compare BEF and dorsolateral foraminotomy (DF) and (3) to provide clinical results of the first client‐owned dog treated by way of BEF.

**Study design:**

Ex vivo cadaveric study, case report.

**Sample population:**

A total of 18 cadaveric lumbosacral spinal specimens (L3–S3). A 4‐year‐old mixed breed dog with lumbosacral foraminal stenosis.

**Methods:**

A surgical technique using a 3.0 mm 30° arthroscope (BEF‐A) or a 1.9 mm 0° needle arthroscope (BEF‐N) was developed in six cadaveric spines. Bilateral L7–S1 foraminotomy was performed in 12 spinal specimens (24 neuroforamina) by (1) DF, (2) BEF‐A or (3) BEF‐N (*n* = 8/group). Visualization, iatrogenic nerve root damage, and foraminal enlargement were compared between the three procedures.

**Results:**

BEF‐A provided superior visualization compared to DF and BEF‐N (*p* < .05). Iatrogenic nerve root damage was not observed in any of the procedures. All procedures resulted in significant enlargement of the neuroforamen (58 ± 33%, *p* < .01). BEF‐A (81.3 ± 30.0%) resulted in significantly more enlargement compared to DF (59.7 ± 33.7%, *p* = .03) and BEF‐N (51.1 ± 38.8%, *p* = .04). BEF‐A was successfully used to treat a client‐owned dog with L7–S1 foraminal stenosis.

**Conclusion:**

BEF‐A provided superior visualization and efficacy compared to DF.

**Clinical significance:**

BEF‐A is a safe and effective surgical technique for treating dogs with L7–S1 foraminal stenosis. The technique should be evaluated in larger clinical studies.

## 1 INTRODUCTION

Degenerative lumbosacral stenosis (DLSS) is a common cause of back pain, neurological deficits and hindlimb lameness in medium‐ to large‐breed dogs.^1^ Lumbosacral foraminal stenosis, which involves compression of the L7 nerve root within the L7‐S1 intervertebral foramen, has been reported in 68% of dogs affected by DLSS.^2^ The intervertebral neurovascular foramen can be divided into an entry, middle and exit zone.^3^ In dogs, compression of the L7 nerve root mainly occurs in the foraminal exit zone.^2^

Dorsolateral foraminotomy as described by Gödde and Steffen is a widely accepted technique to treat L7–S1 foraminal stenosis.^2^ Despite its high success rate (good to excellent outcome in terms of neurological grade and limb function in 95% of the cases), this open procedure may provide limited visualization, leading to incomplete decompression or iatrogenic nerve injury.^2^ Furthermore, it requires an extensive, open approach, increasing the risk of morbidity.

In human medicine, minimally invasive spinal surgery has gained traction due to its potential to improve decompression accuracy, reduce iatrogenic injuries and postoperative morbidity, and facilitate faster rehabilitation.^4^, ^5^, ^6^, ^7^, ^8^, ^9^, ^10^, ^11^ In veterinary medicine, minimally invasive techniques have been described using specialized equipment.^3^, ^12^, ^13^, ^14^, ^15^, ^16^, ^17^, ^18^, ^19^, ^20^, ^21^ However, such equipment is often costly and may not be readily available to most veterinary practitioners.

Recently, a minimally invasive lumbosacral foraminotomy using dedicated endoscopic equipment has been described.^22^ However, to date, a minimally invasive technique for L7–S1 foraminotomy in dogs that relies exclusively on readily available, arthroscopic equipment has not been reported. Additionally, a comparison between endoscopic and conventional dorsolateral foraminotomy (DF) in terms of visualization, efficacy, and safety has not been reported.

The objectives of this study were to (1) Develop a biportal endoscopic foraminotomy (BEF) technique for L7–S1 using commonly available, arthroscopic equipment. (2) Compare BEF with DF regarding surgical time, tissue trauma, visualization, iatrogenic nerve injury, and foraminal enlargement. (3) Report the clinical application of BEF in a dog with L7–S1 foraminal stenosis. We hypothesized that (1) BEF using arthroscopic equipment is feasible for L7–S1 in dogs. (2) BEF is equally safe, provides improved visualization and offers greater foraminal enlargement than DF. (3) BEF is applicable in a clinical case with L7–S1 foraminal stenosis as a proof of concept.

## 2 MATERIALS AND METHODS

### 2.1 Ex vivo study (Phases I and II)

#### 2.1.1 Specimen collection

A total of 18 lumbosacral spinal specimens (L3–S3) were collected from medium‐ to large‐sized dogs euthanized for reasons unrelated to spinal disease (Table S1). Informed owner consent for scientific use of the specimens was obtained in all cases. The dogs weighed between 18.4 and 40.8 kg (median, 30.7 kg) and ranged in age from 2 to 15 years (median, 9.5 years). The specimens were harvested with the overlying skin and soft tissues preserved, were wrapped in saline‐soaked surgical swabs and stored at −20°C. Before use, they were thawed at room temperature for 24 h.

#### 2.1.2 Phase I: Establishment of the BEF technique

Six spinal specimens (*n* = 12 neuroforamina) were used. Phase I aimed to develop the surgical technique and to assess and optimize different approaches, working channels, arthroscopic instrumentation, and ergonomics while maintaining minimally invasive principles.

**FIGURE 1:**
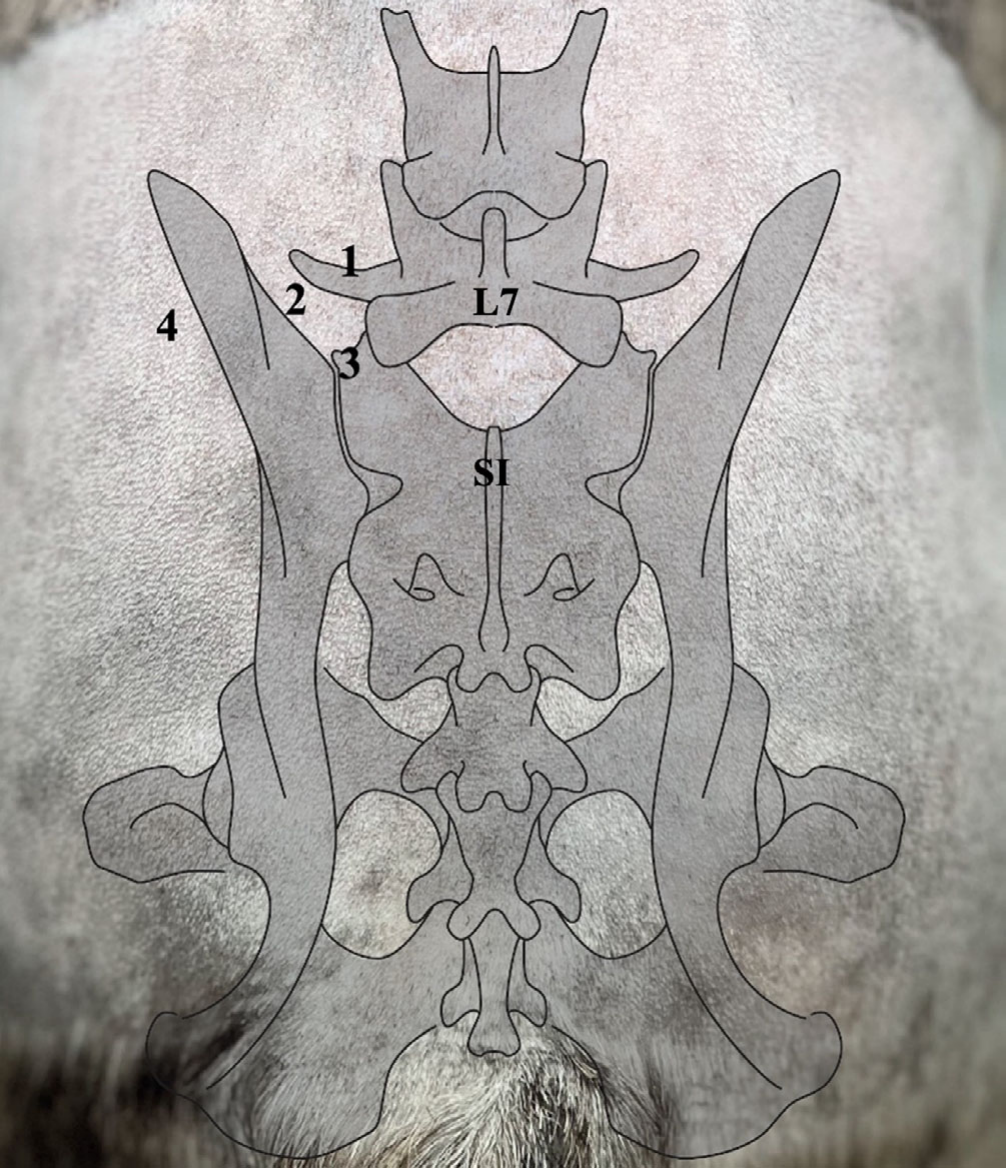
Different camera ports and working channels evaluated during Phase I. (1) Cranial port, situated at the base of the L7 transverse process (TP). (2) Craniolateral port, situated 1 cm lateral to the base of the L7 TP. (3) Caudal port, situated at the cranial edge of the sacroiliac joint. (4) Lateral transiliac port, 1 cm caudal to the level of L7 TP.

1. *Camera port and working channels*
  The following port locations were tested (Figure 1):
  - Cranial port, situated at the base of the L7 transverse process
  - Craniolateral port, situated 1 cm lateral to the base of the L7 transverse process
  - Caudal port, situated at the sacroiliac junction
  - Lateral transiliac port

2 *Arthroscopes*. Two types of arthroscopes were evaluated:
  - 3.0 mm 30° oblique arthroscope, 138 mm long (Arthrex Vet Systems, Naples, Florida)
  - 1.9 mm 0° NanoNeedle Scope, 125 mm (Arthrex)
3 *Arthroscopic working cannulas*:
  - Low profile cannulas, 5 mm (Arthrex)
  - PassPort Button cannulas, 6 mm and 8 mm (Arthrex)
4 *Synergy resection shaver handpiece* (Arthrex), attached to water pump (DualWave arthroscopy pump, Arthrex), and the following shaver attachments/tips:
  - Torpedo shaver blade, 3.5 mm (Arthrex)
  - Dissector shaver blade, 3.5 mm (Arthrex)
  - SabreTooth shaver blade, 3.5 mm (Arthrex)
  - Oval and round burrs, 3.0 mm (Arthrex)
  - ClearCut burr, round, 4.0 mm (Arthrex)
5 *Miscellaneous instruments*:
  - Kerrison rongeurs (1–3 mm with standard or reverse tips)
  - Nerve hook retractors (small or large, right‐angled round retractors)
  - Periosteal elevators (Freer dissector, Adson round periosteal elevator)

To adhere to minimally invasive surgical principles, intraoperative fluoroscopy (Veradius Neo 718 131 Mobile C‐Arm, Philips, Netherlands) was used at key stages of the procedure.

#### 2.1.3 Phase II: Evaluation and comparison of BEF with conventional DF

A total of 12 lumbosacral specimens (*n* = 24 neuroforamina) were used in Phase II. Bilateral foraminotomies were performed in each specimen using one of the following surgical techniques (*n* = 8 neuroforamina per technique):

1. Conventional, open DF as described in previous literature.^2^ Briefly, a dorsolateral approach to the lumbosacral junction using blunt and sharp dissection was performed to expose the L7 transverse process and pedicle. A dorsolateral L7–S1 foraminotomy was performed using a high‐speed drill (ELAN 4 Power System [Aesculap, Inc., Tuttlingen, Germany]) to remove bone from the dorsal aspect of the L7 transverse process to the base of the cranial articular process and into the foraminal exit zone, opening the lateral spinal canal and intervertebral foramen. Decompression was extended as needed with fine burrs (2.0–4.0 mm) and 2 mm Ferry‐Smith‐Kerrison rongeur, while protecting the nerve root. The procedure was considered complete when the nerve root was fully decompressed and freely mobile several millimeters laterally and cranially without impingement.
2. Minimally invasive – biportal endoscopic foraminotomy, using a 3.0 mm 30° arthroscope (BEF‐A), as established in Phase I.
3. Minimally invasive – biportal endoscopic foraminotomy, using a NanoNeedle Scope 125 mm (BEF‐N), as established in Phase I.

The choice of surgical technique for each neuroforamen was randomized using a number generator. Each technique was applied equally, with two different techniques used per specimen to allow intraspecimen comparison. The DF procedures were performed by a board‐certified veterinary neurologist (FS), while the BEF procedures were carried out by a board‐certified veterinary surgeon (LS). Pre‐ and postoperative computed tomography (CT) scans (IOon, Philips AG, Zurich, Switzerland) were acquired for all specimens and were used to quantitatively assess the outcomes of the three surgical procedures.

#### 2.1.4 Outcome measures

The following parameters were assessed postoperatively by an independent observer (DB):

1. *Surgical time*: Divided into approach time – time taken to visually or arthroscopically identify the base of the L7 transverse process – and foraminotomy time – time from initiation of bone removal to completion of the procedure.
2. *Incision length*: The lengths of the skin and fascia incisions were recorded separately. Incision length was measured using a surgical marker (Securline Surgical Skin Marker, Aspen Surgical Products, Caledonia, Michigan). For BEF procedures, the length of skin and fascia incisions were considered identical as the approach was performed through a stab incision.
3. *Visualization of the L7 nerve root*: Nerve root visualization was graded as follows:
  - Good: the post‐ganglionic, ganglion, and preganglionic portions of the nerve root could be identified
  - Intermediate: the ganglion and post‐ganglionic portions of the nerve root could be identified
  - Poor: the post‐ganglionic portion of the nerve root or less could be identified

For BEF procedures, intraoperative endoscopic images were used for assessment; for DF, high‐definition photographs via the incision were used.

4 *Visualization of the neuroforaminal zones*: Based on previous literature,^3^ each neuroforamen was divided into exit, middle and entry zones. Visualization of each zone was graded as:
  - Good: the anatomical boundaries could be completely identified
  - Intermediate: the anatomical boundaries could be partially identified
  - Poor: the anatomical boundaries could not be identified
5 *Presence of macroscopic iatrogenic nerve root damage*: All the nerve roots were inspected for iatrogenic damage (yes/no). Iatrogenic nerve root damage was assessed following removal of all soft tissue surrounding the L7–S1 neuroforamen, preserving the L7 nerve root.
6 *Assessment of procedure efficacy*: Procedure efficacy was evaluated using pre‐ and postoperative CT scans by an independent observer (DB). The volume (mm^3^) of the L7–S1 neuroforamen was calculated pre‐ and postoperatively using previously described methods.^23^ The difference in volume was used to calculate the extent of foraminal enlargement for DF, BEF‐A, and BEF‐N.

In addition, the postoperative CT images were qualitatively assessed with respect to bone removal in different foraminal zones. Bone removal was assessed for the following anatomical regions:

- Region dorsal to foramen: the caudal articular process of L7, facet joint, and cranial articular process of S1
- Region ventral to foramen: the cranioventral border of the foramen at the base of the L7 transverse process (“cranioventral lip”), the vertebral body of L7 and the L7–S1 intervertebral disc
- Region cranial to foramen: the pedicle of L7
- Region caudal to the foramen: the pedicle of S1

For each foraminotomy, bone removal in each region was assessed as follows by one observer (DB) in blinded fashion:

- Appropriate: sufficient bone removal in targeted regions
- Insufficient: insufficient bone removal in planned periforaminal regions
- Excessive: excessive bone removal in planned regions or bone removal in unplanned regions

An appropriate foraminotomy was defined as follows:

- Dorsoventral extension: from the dorsal aspect of the transverse process of L7 to the base of the caudal articular process of L7.
- Craniocaudal extension: from the caudodorsal origin of the transverse process into the exit zone of the intervertebral foramen.

#### 2.1.5 Data analysis

The data generated in Phase II of the study were analyzed using descriptive and inferential statistics. For continuous variables, bar graphs and box plots were created to identify spurious observations and assess data distributions using commercially available software (GraphPad Prism version 10.0.0 for macOS, GraphPad Software, Boston, Massachusetts; www.graphpad.com). For nominal variables, parameter scores/grades were tabulated and grouped according to procedure (DF, BEF‐A, BEF‐N).

Inferential analyses were conducted using R statistical software.^24^ Outcome measures were compared across the three procedures (DF, BEF‐A, BEF‐N). Nominal variables were analyzed using Fisher's exact test. Continuous variables were analyzed using linear mixed models. The models included “surgical procedure” (BEF‐A, BEF‐N, DF) as a fixed effect and “spinal specimen” as a random effect to account for repeated measures within each specimen. The Benjamini–Hochberg correction was applied to adjust for multiple comparisons. *p* < .05 was considered statistically significant.

### 2.2 Phase III: Case report/clinical proof of concept

A 3.5‐year‐old female spayed mixed‐breed dog (bodyweight: 18.9 kg) was referred to the Clinic for Small Animal Surgery, Vetsuisse Faculty, Zurich, Switzerland, with a history of lumbar pain and progressive difficulty jumping. The dog exhibited a grade 1/4 lameness of the right hindlimb and pain on palpation of the lumbar/lumbosacral region. Magnetic resonance imaging of the lumbosacral spine (Philips Ingenia 3 T, Philips AG, Zürich, Switzerland) revealed right‐sided foraminal stenosis with moderate L7 neuropathy and moderate to marked stenosis of the exit zone of the L7–S1 neuroforamen.

Following an initial phase of clinical improvement with conservative treatment, recurrence of clinical signs was observed over a period of 10 months. Consequently, surgical treatment was recommended. The owner elected to proceed with minimally invasive BEF‐A. Written informed consent was obtained, including agreement to convert to open DF if deemed necessary by the primary surgeons.

The dog's preoperative neurologic status was systematically assessed by a board‐certified veterinary neurologist (FS) using a standardized grading scheme for DLSS patients,^25^ yielding a total score of 17/21. Surgery was performed via right‐sided BEF‐A. Endoscopic images were collected, and intraoperative complications and surgical time were recorded.

Postoperative follow‐up included clinical examinations and DLSS grading at 2 weeks and 6 months, and postoperative magnetic resonance imaging (MRI) of the lumbosacral junction at 6 months.

## 3 RESULTS

### 3.1 Phase I: Establishment of surgical technique for BEF

#### 3.1.1 Approaches and camera/working channels

Establishment of the following camera/instrument ports was deemed most efficient and ergonomic (Figure 1):

1. Dorsal keyhole port, situated at the caudal border of the base of the L7 transverse process.
2. Caudal port, situated at the cranial border of the sacroiliac junction.

Other ports were not deemed effective or ergonomic, as the L7–S1 neuroforamen could not be adequately visualized or approached. A transiliac approach was not considered suitable, as movement of the endoscope and instruments was significantly restricted through the transiliac window, thereby complicating the surgical procedure. Arthroscopic working cannulas were not incorporated into the standardized procedure because they resulted in crowding and reduced the efficacy of instrument control.

#### 3.1.2 Instrumentation

A 3.0 mm, 30° oblique arthroscope (138 mm length; Arthrex Vet Systems) and a 1.9 mm, 0° NanoNeedle Scope (125 mm; Arthrex) were used.

For surgical debridement of soft tissues, a Torpedo Blade 3.5 mm shaver tip (Arthrex) was selected. Other shaver tips, including a 3.5 mm Dissector shaver blade and the 3.5 mm SabreTooth shaver blade (Arthrex), were considered subjectively too aggressive for soft tissue debridement, with a potentially increased risk of iatrogenic nerve root damage. Therefore, these shaver tips were not used for this purpose.

For bone removal, a Round Burr 3.0 mm (Arthrex) and ClearCut Burr, round, 4.0 mm (Arthrex) were selected. The rounded shape of these shaver tips allowed accurate and precise bone removal in the foraminal exit and middle zones. Oval burr shaver tips were excluded because the configuration and shape of the burring head did not allow adequate bone removal. With respect to rongeurs, only the 1 mm Kerrison rongeurs were ultimately selected for the final stages of the procedure. This choice was based on instrument size, as the 2–3 mm rongeurs proved difficult to introduce into the foraminotomy defect and to handle effectively in this setting. The Kerrison rongeur was used only in the final step to achieve targeted removal of small bone fragments; therefore, the 2 and 3 mm instruments were considered too large for this purpose.

Irrigation pressure and flow: The choice of irrigation parameters was guided primarily by evidence from the human spinal endoscopy.^26^, ^27^ Human studies evaluating biportal and interlaminar endoscopic spinal procedures have demonstrated that low or conservative irrigation pressures (<30 mmHg) are associated with smaller increase in epidural and intracranial pressures and a significantly reduced risk of pressure‐related complications.^26^, ^27^ Based on these findings, an irrigation pressure of 15 mmHg was selected, representing a conservative setting well below commonly reported thresholds while still allowing adequate visualization and effective debris removal.

#### 3.1.3 Challenges/complications

Several technical challenges and complications were encountered during establishment of the procedure, primarily related to orientation, landmark identification, and bone removal. These included operating at the wrong level (L6–L7), incorrect identification of the transverse process, and an incorrect starting point for drilling. All these emphasized the need for routine fluoroscopic confirmation of key anatomical landmarks, such as the L7 transverse process and cranial border of the sacroiliac joint and sufficient soft tissue removal surrounding these landmarks and the L7–S1 neuroforamen. Additional challenges involved incorrect drilling direction, which could result in excessive removal in a cranial (L7 pedicle and transverse process), ventral (L7 vertebral body), or dorsal (articular processes and facet joint) direction; in such situations, guidance with a nerve hook, which was intermittently placed into the neuroforamen, was required by following its course. Lastly, premature use of a Kerrison rongeur was also identified as a risk for sharp nerve root injury, supporting the recommendation to continue drilling for as long as possible before its use.

#### 3.1.4 Surgical procedure

**FIGURE 2:**
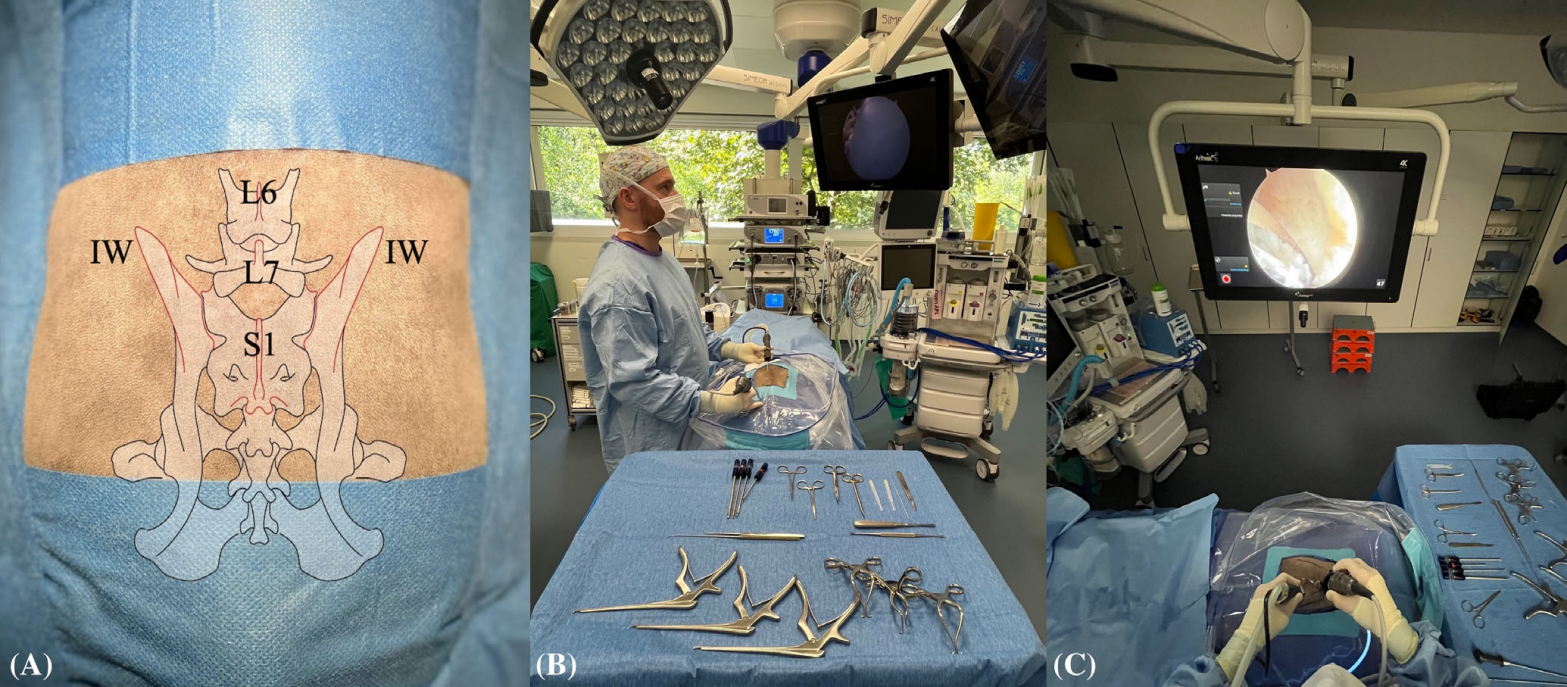
(A) Macroscopic view of a lumbosacral specimen prepared for surgery. The desired surgical field is highlighted within the square. Bony landmarks are highlighted in red and include the cranial aspect of the iliac wing (IW) and the spinous processes of L6, L7, and S1. (B) Full view of the surgical setup, showing the surgeon positioned lateral to the patient on the side of the foraminotomy. (C) Point of view of the surgeon. IW, iliac wing.

**FIGURE 3:**
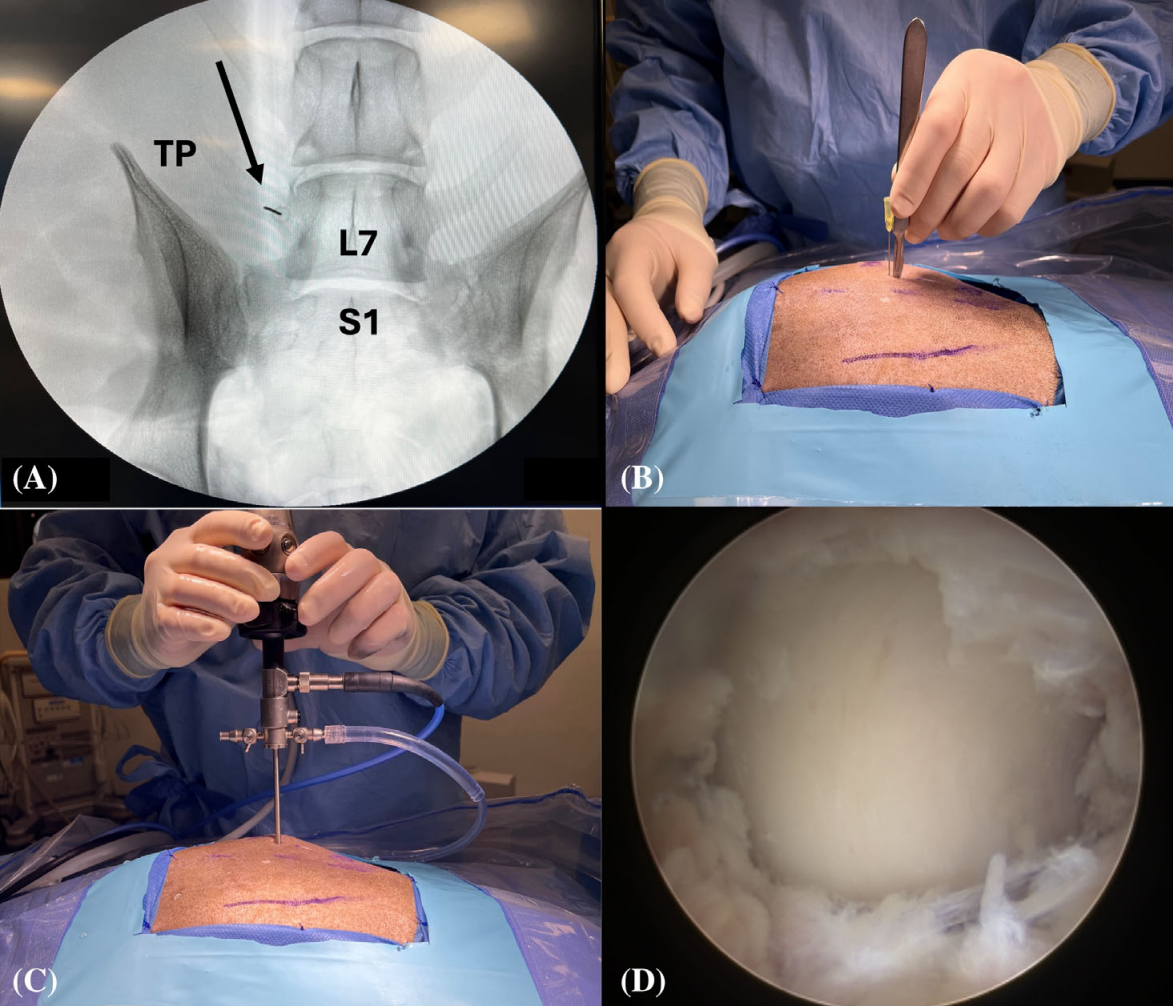
Establishment of the keyhole port. Fluoroscopic confirmation of landmarks. (A) Hypodermic needle is positioned on the base of the L7 transverse process (TP; black arrow). (B) Stab incision parallel to the hypodermic needle to establish the keyhole port. (C) Insertion of the scope into the keyhole. (D) Endoscopic image of the base of the TP before soft tissue debridement. In the macroscopic images, iliac wings and the dorsal midline have been marked with a skin marker. In all images, cranial is left and dorsal is up.

**FIGURE 4:**
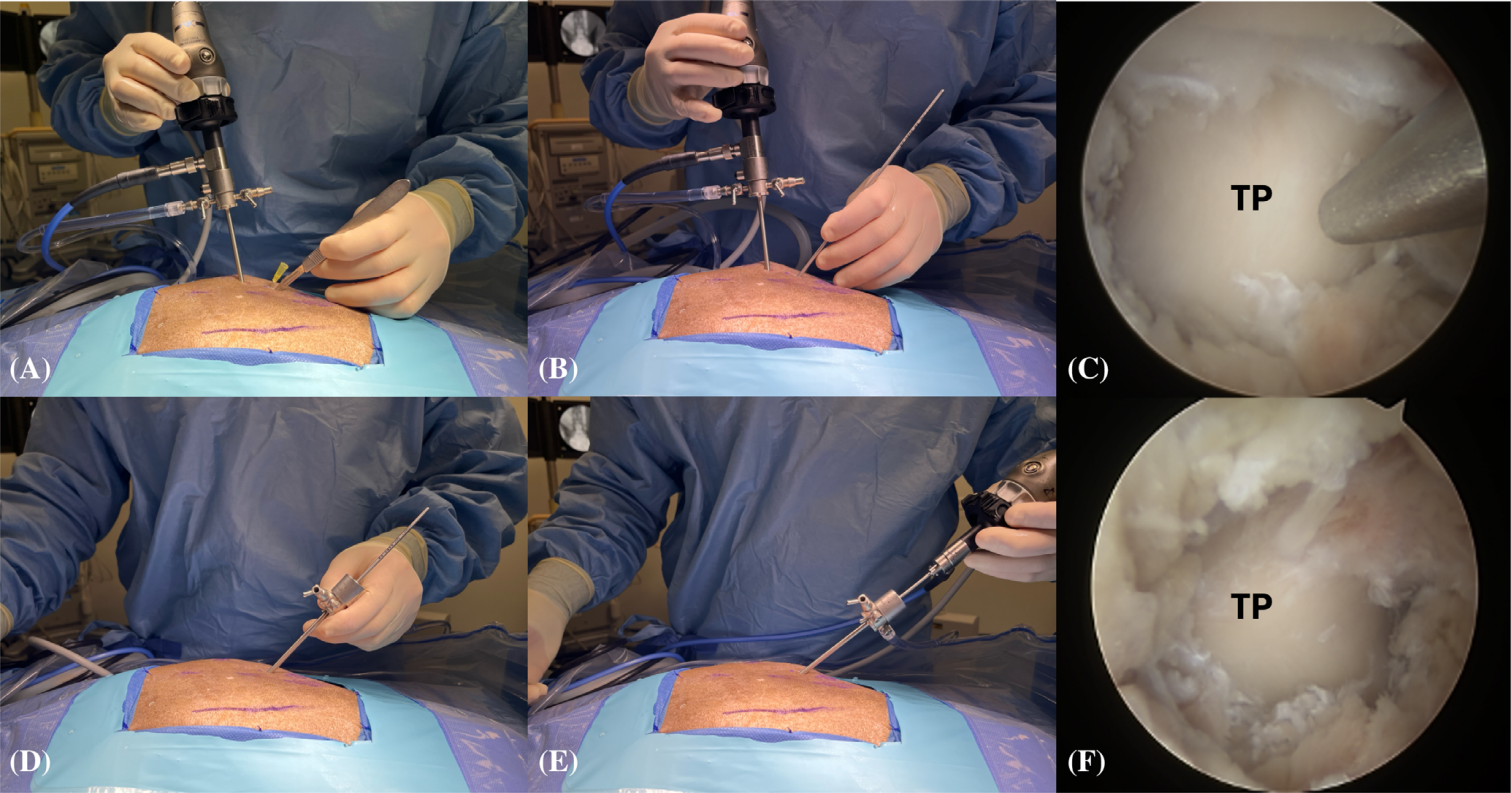
Establishment of the caudal port using the switching stick technique. (A) Stab incision using a hypodermic needle at the cranial edge of the sacroiliac joint, directed toward the base of the L7 transverse process (TP) under endoscopic guidance. (B) Insertion of the switching stick through the stab incision. (C) Endoscopic image of the switching stick placed on the base of the L7 TP. (D–E) Switching stick procedure, with the camera moved from the keyhole port to the caudal port. (F) Endoscopic view of the base of the L7 TP from the caudal port. Left is cranial and up is dorsal in all images.

**FIGURE 5:**
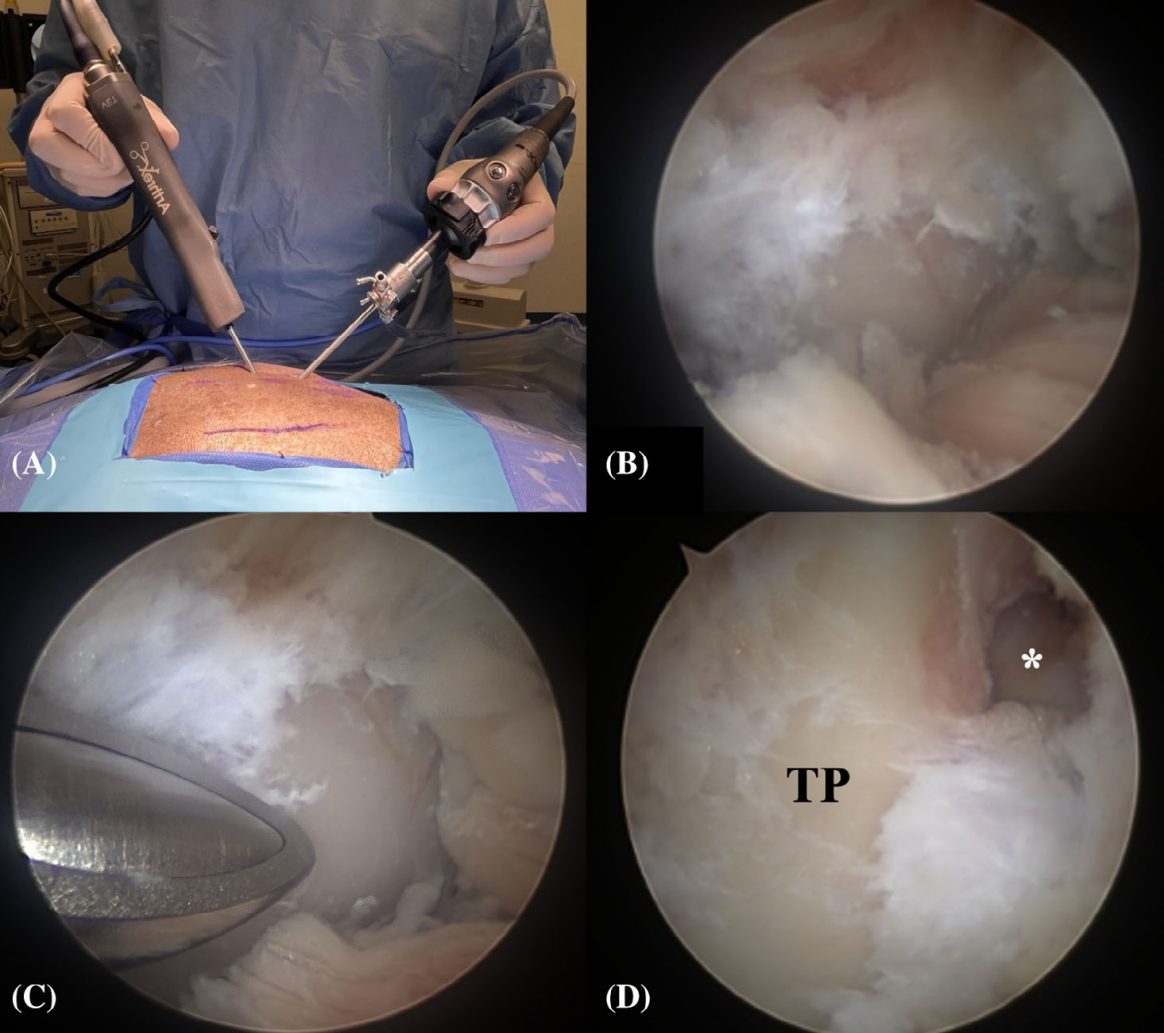
Periforaminal soft tissue debridement. (A) Macroscopic image of the torpedo shaver (Arthrex) in the dorsal keyhole. (B) Endoscopic image before soft tissue debridement. (C) During debridement. (D) After debridement. (E) Exit zone of the neuroforamen (*) after completed debridement. Left is cranial and up is dorsal. TP, L7 transverse process.

**FIGURE 6:**
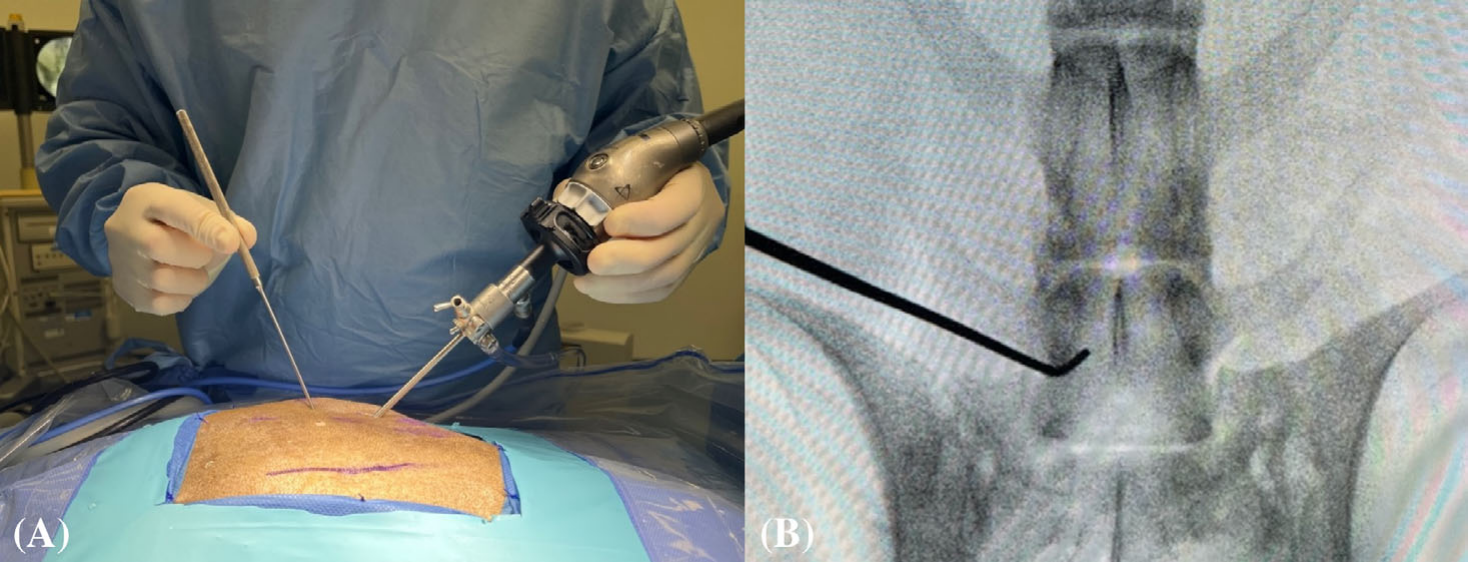
Confirmation of foraminotomy location. (A) Macroscopic image of a nerve hook inserted through the keyhole port. (B) Fluoroscopic image showing the hook in the L7–S1 neuroforamen. In the macroscopic image, left is cranial and up is dorsal.

**FIGURE 7:**
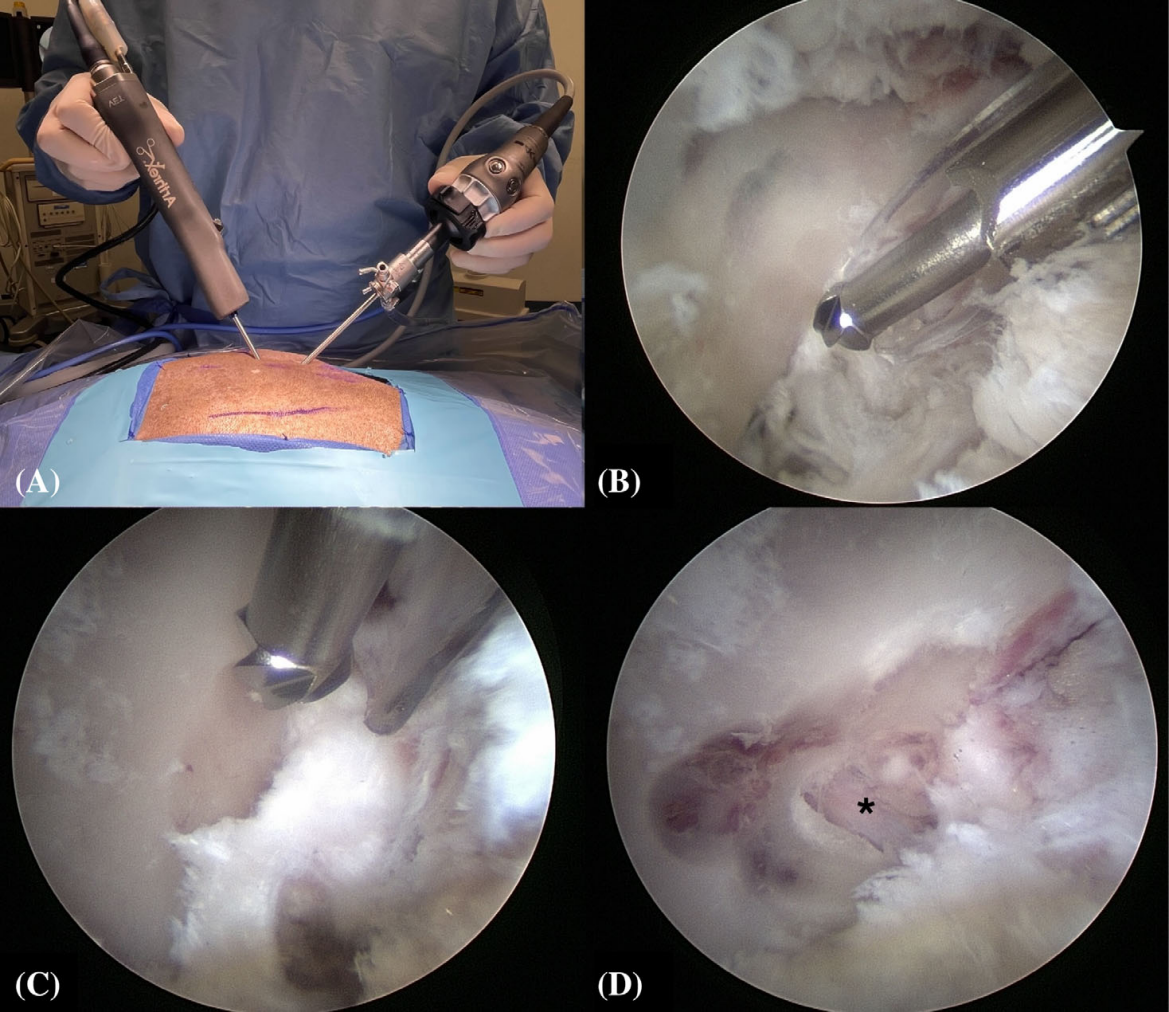
(A) Macroscopic image of the shaver inserted through the keyhole port. (B) ClearCut Round Burr 4 mm (Arthrex) on the caudal aspect of the L7 transverse process base. (C) Burring in a craniodorsal direction. (D) Incomplete foraminotomy showing the L7 nerve root (*). Left is cranial and up is dorsal.

**FIGURE 8:**
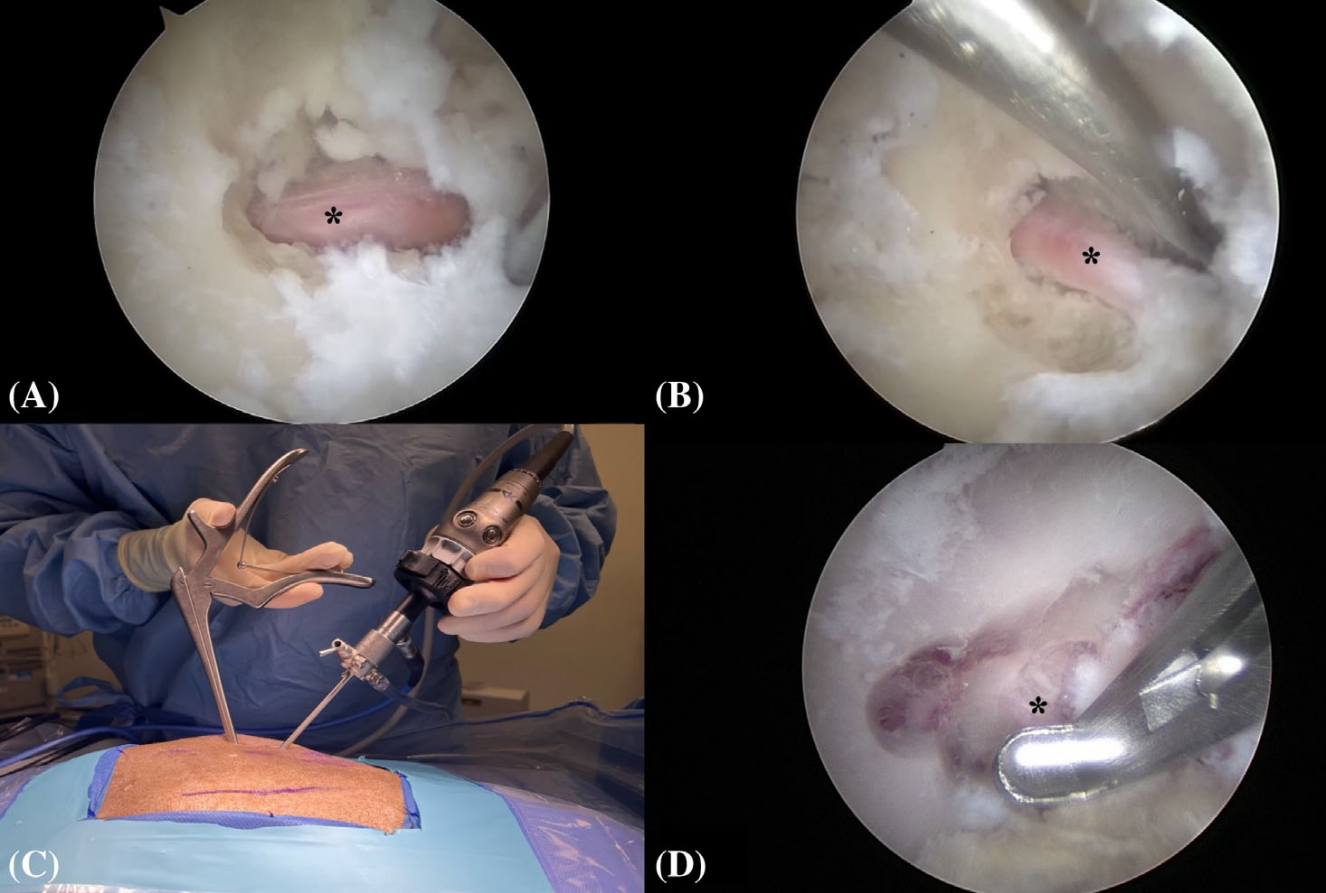
Visual confirmation of the L7 nerve root (*) and foraminotomy with a Kerrison rongeur. (A) Nerve root visible without retraction after burring. (B) Gentle retraction using a nerve retractor. (C) Insertion of a 1 mm Kerrison rongeur through the keyhole port. (D) Endoscopic image showing rongeur extending the foraminotomy craniodorsally. Left is cranial and top is dorsal.

**FIGURE 9:**
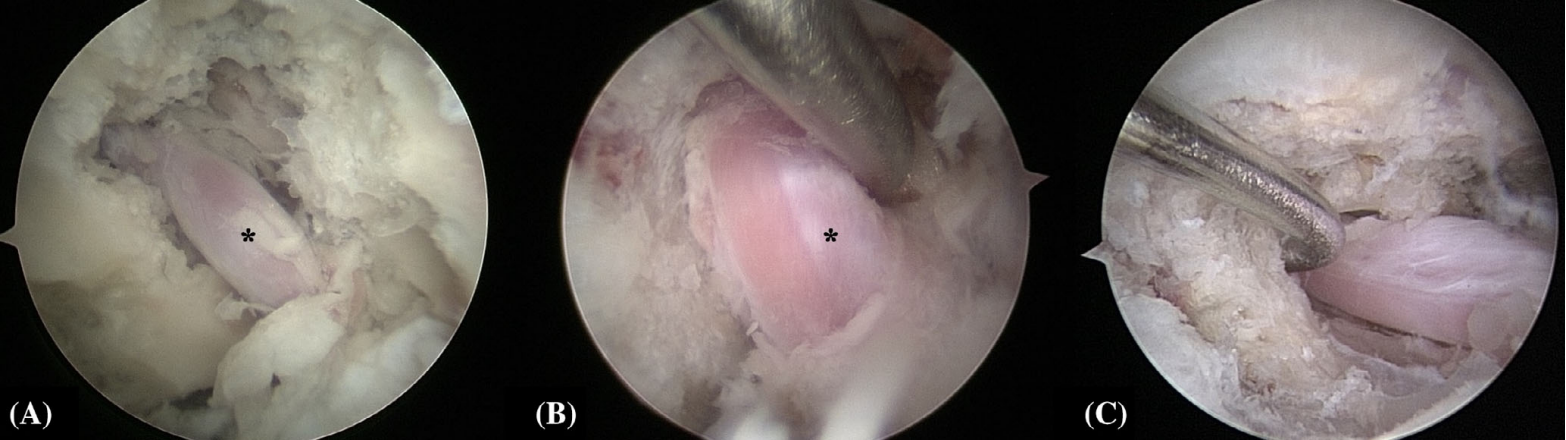
Completion of foraminotomy. (A–C) Endoscopic views of the nerve root in the neuroforamen, free of impingement. (B) Nerve mobility within the enlarged exit and middle zones assessed using a retractor. Note the dorsal ganglion (black asterisk).

The established procedure for BEF involved the following steps (detailed surgical description and complete instrumentation list: Supplementary Files S1 and S2):

- Step 1: The patient was positioned in sternal recumbency, and the surgeon stood lateral to the patient on the side requiring foraminotomy (Figure 2A,B). The following surgical landmarks were identified:
  - Craniodorsal aspect of the iliac wing
  - Dorsal spinous processes of L6, L7, and S1 vertebrae
- Step 2: The base of the L7 transverse process was identified using a hypodermic needle (20 gauge × 2¾) (0.9 mm × 70 mm) under fluoroscopic control (Figure 3). Similarly, the position of the cranial edge of the sacroiliac joint was identified.
- Step 3: Establishment of the keyhole port: A 1 cm stab incision (craniocaudal direction) was made through the skin, subcutis, and lumbosacral fascia, directed parallel to the hypodermic needle inserted into the base of the L7 transverse process (TP), using a No. 11 scalpel blade (Figure 3B). The arthroscope was inserted through the incision to visualize the base of the L7 TP (Figure 3C,D). Sterile Ringer's lactate solution (Fresenius Kabi, Lake Zurich, Illinois) was continuously infused through the arthroscopic sheath using the DualWave Arthroscopy Fluid Management System (Arthrex) at a pressure of 15 mmHg to expand the endoscopic working space and enhance surgical visualization. Continuous egress of fluid from the surgical field was maintained through the keyhole port, which, due to its width, allowed ongoing outflow of excess fluid.
- Step 4: Establishment of the caudal port: A stab incision was performed similar to Step 3, parallel to the hypodermic needle inserted at the sacroiliac joint (Figure 4A). Using a switching stick technique, the endoscope was removed from the keyhole incision and reinserted into the caudal incision (Figure 4B–4E). The base of the TP could now be visually confirmed from the caudal port (Figure 4F).
- Step 5: Soft tissue debridement: The arthroscopic power shaver handpiece (APS II Shaver Handpiece, Arthrex) was inserted through the dorsal keyhole port (Figure 5A). Debridement of the periforaminal soft tissue structures (Figure 5B) surrounding the caudal aspect of the base of the L7 TP was performed under continuous suction using a Torpedo Blade Shaver Tip 3.5 mm (Arthrex) (Figure 5C; Supplementary Video S1), until the boundaries of the neuroforamen were clearly identified (Figure 5D).
- Step 6: Correct localization was confirmed by inserting a nerve hook retractor into the L7–S1 neuroforamen and performing a fluoroscopic radiograph (Figure 6A,B).
- Step 7: Foraminotomy was initiated at the caudal aspect of the L7 transverse process in a craniodorsal direction using a ClearCut Round Burr shaver tip (Size: 4 mm, Arthrex) inserted through the dorsal keyhole incision (Figure 7A–D). Choosing where to initiate burring was facilitated by accurate soft tissue preparation of periforaminal soft tissues and soft tissue removal from the lateral pedicle wall. The direction of burring was guided by identifying the direction of the neuroforamen by way of intermittent probing it with the nerve hook.
- The transparent protective sheath of the shaver tip provided additional protection to the L7 nerve root as the foraminotomy progressed cranially (Supplementary Video S2).
- Step 8: After identification of the L7 nerve root, the foraminotomy was expanded cranially and medially using a smaller‐sized 3 mm arthroscopic burr and 1 mm Kerrison rongeurs (Figure 8A–D; Supplementary Video S3).
- Step 9: Determination of endpoints (Figure 9A–C). The procedure was considered complete when:
  - The nerve root was free of any cranial, ventral, and lateral impingements and could be moved freely after gentle traction using the nerve hook retractor
  - The dorsal root ganglion was identified and could be moved freely out of the neuroforamen (Supplementary Video S4)
- Step 10: Closure was performed in routine fashion by suturing the lumbosacral fascia (Monoplus 3–0, B. Braun Surgical S.A., Rubí, Spain), the subcutis (Monosyn 3–0, B. Braun), and the skin (Supramid 3–0, B. Braun) with single interrupted sutures.

### 3.2 Phase II: Assessment and comparison between DF and BEF

All described procedures (DF, BEF‐A, and BEF‐N) were successfully performed on the canine cadaveric specimens, with one exception: left‐sided foraminotomy of specimen No. 6, assigned to the DF group, was excluded due to the presence of a lumbosacral transitional vertebra.

#### 3.2.1 Surgical time

Surgical time (mean ± SD) for DF (27.6 ± 10.7 min) was significantly shorter than for BEF‐A (94.0 ± 31.8 min) and BEF‐N (93.7 ± 25.4 min) (*p* < .01). BEF‐N and BEF‐A were comparable with respect to surgical time (*p* = 1.00).

#### 3.2.2 Incision length

BEF resulted in significantly smaller surgical incisions than DF. DF required a single approach (skin: 71.9 ± 8.6 mm; fascia: 57.0 ± 8.2 mm; Figure 11). BEF‐A and BEF‐N resulted in two separate, significantly smaller incisions (*p* < .01). The keyhole approach required an incision of 10.6 ± 2.7 mm, while the caudal port required an incision of 7.5 ± 3.1 mm.

#### 3.2.3 Visualization of the L7 nerve root

Visualization of the nerve root was significantly better with BEF‐A (*p* < .01) and BEF‐N (*p* = .04; Table 1) compared to DF. Direct visualization of the nerve root through the DF approach was considered poor in most cases (75.0%), while for BEF‐A and BEF‐N, visualization was graded as good in 87.5% and 85.7% of cases, respectively (Figure 12A–J).

**TABLE 1:** Assessment of nerve root visualization for dorsolateral foraminotomy (DF), biportal endoscope‐assisted foraminotomy with a 3.0 mm 30° oblique arthroscope (BEF‐A) and biportal endoscope‐assisted foraminotomy with a Nanoneedle (BEF‐N).

	Visualization of the nerve root		
	Good	Intermediate	Poor
DF	0% (0/8)	25% (2/8)	75% (6/8)
BEF‐A	87.5% (7/8)	12.5% (1/8)	0% (0/8)
BEF‐N	85.7% (6/7)	0% (0/7)	14.2% (1/7)

#### 3.2.4 Visualization of neuroforaminal zones

**FIGURE 10:**
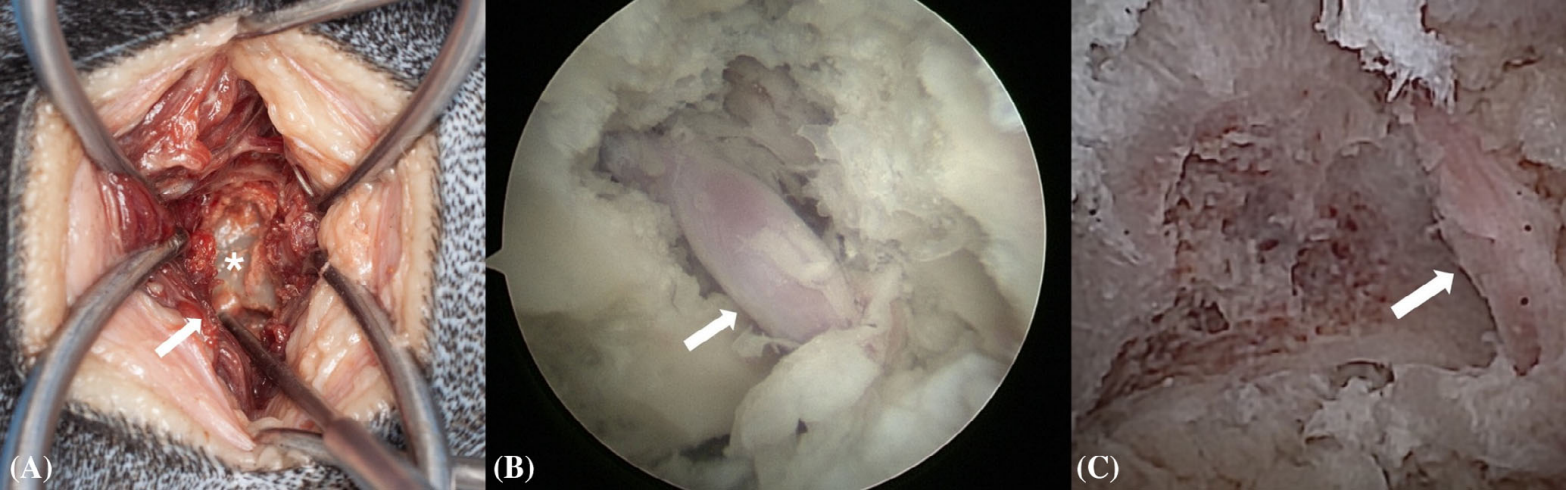
“Best view” of the L7 nerve root (white arrows) via. (A) Traditional dorsolateral foraminotomy (asterisk = L7 transverse process). (B) BEF using a 3.0 mm arthroscope. (C) BEF using the nanoneedle scope. Note the need for nerve retraction in (A).

Visualization of the foraminal exit zone was considered good for DF (50.0%), BEF‐A (100%), and BEF‐N (57.1%; Table 2), with no significant differences between groups (*p* > .17; Figure 12E–J). Direct visualization of the middle zone was graded significantly higher for BEF‐A (*p* < .01) and BEF‐N (*p* < .01) compared to DF, with good visualization in 50.0%, 100%, and 85.7% of cases, respectively (Figure 10). Visualization of the entry zone was considered poor in most procedures for DF (100%), BEF‐A (75.0%), and BEF‐N (85.7%; *p* > .67).

**TABLE 2:** Assessment of foraminal zone visualization for dorsolateral foraminotomy (DF), biportal endoscope‐assisted foraminotomy with a 3.0 mm 30° oblique arthroscope (BEF‐A) and biportal endoscope‐assisted foraminotomy with a Nanoneedle (BEF‐N).

	Visualization of the foraminal zones		
	Good	Intermediate	Poor
Exit zone			
DF	50% (4/8)	50% (4/8)	0% (0/8)
BEF‐A	100% (8/8)	0% (0/8)	0% (0/8)
BEF‐N	57.1% (4/7)	42.9% (3/7)	0% (0/7)
Middle zone			
DF	0% (0/8)	50% (4/8)	50% (4/8)
BEF‐A	100% (8/8)	0% (0/8)	0% (0/8)
BEF‐N	85.7% (6/7)	14.2% (1/7)	0% (0/7)
Entry zone			
DF	0% (0/8)	0% (0/8)	100% (8/8)
BEF‐A	0% (0/8)	25.0% (2/8)	75% (6/8)
BEF‐N	0% (0/7)	14.2% (1/7)	85.8% (6/7)

**FIGURE 11:**
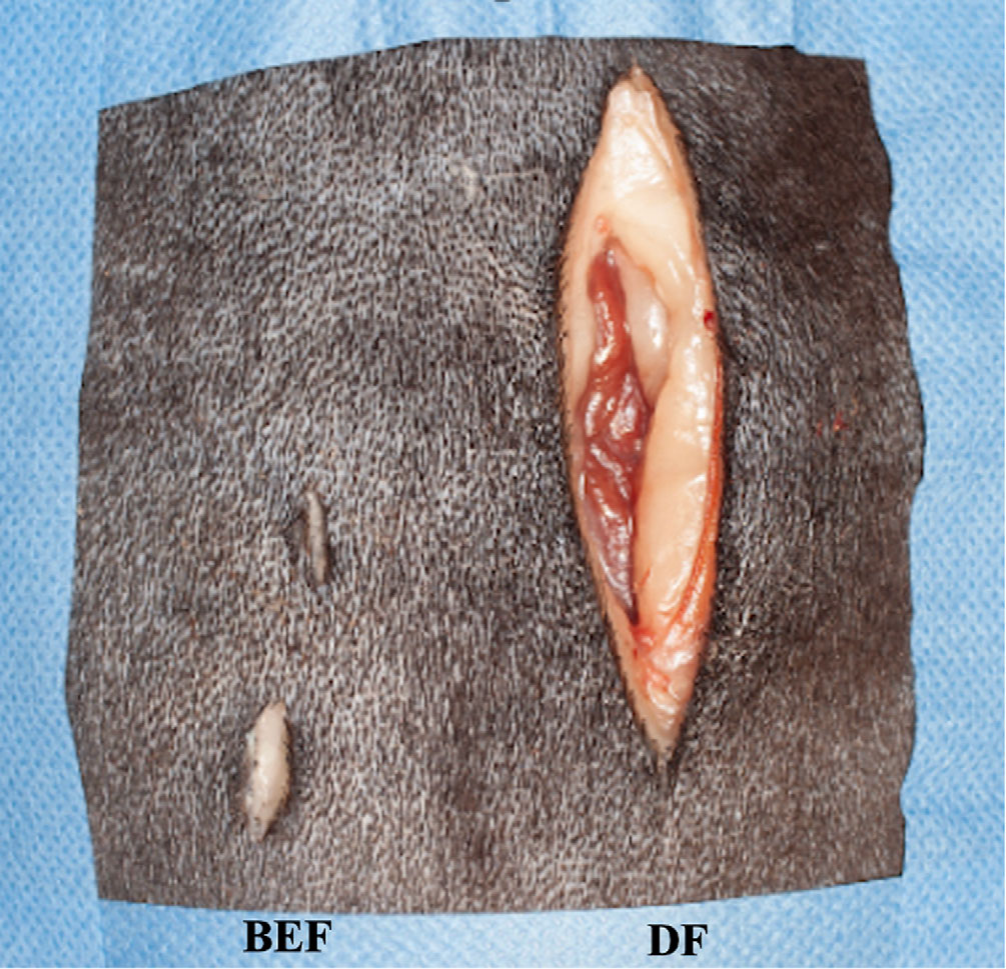
Macroscopic view of the surgical approach for biportal endoscopic foraminotomy (left) and open dorsolateral foraminotomy (right). Note the marked difference in approach.

**FIGURE 12:**
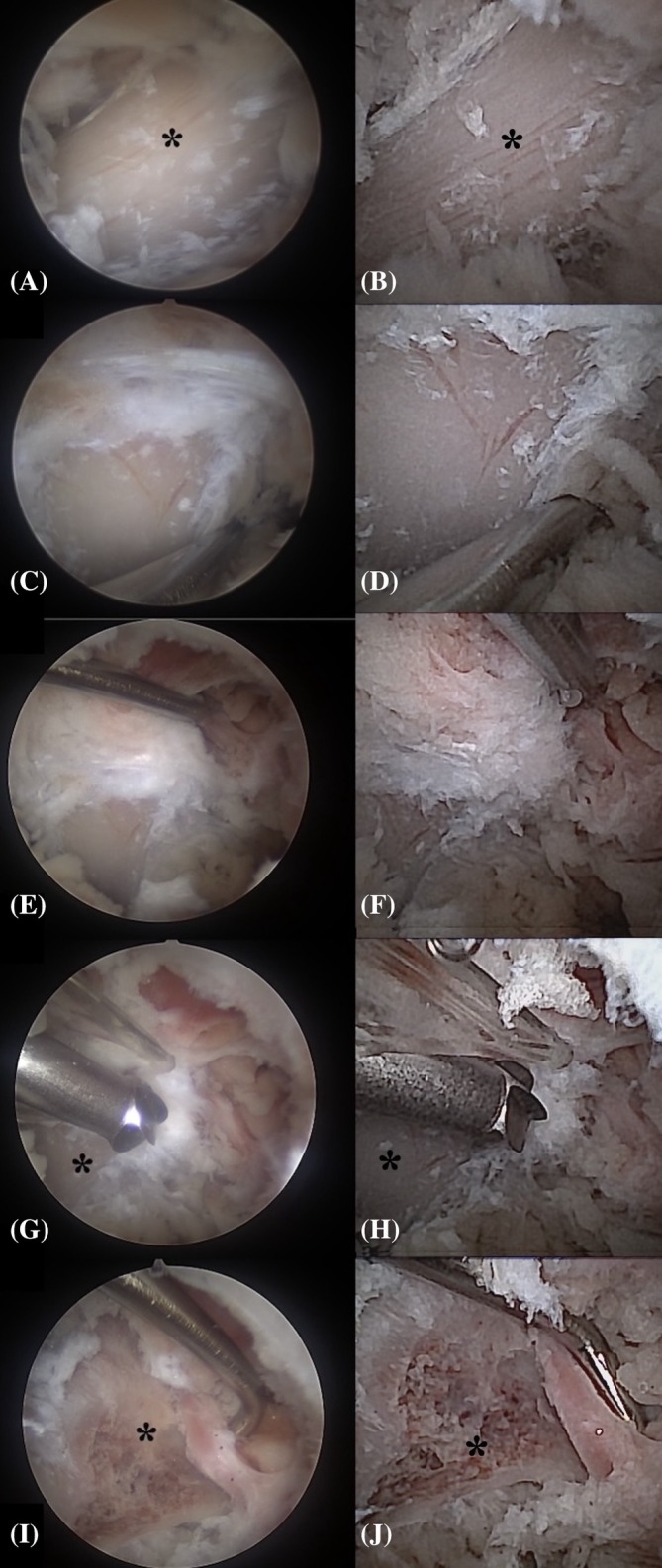
Comparative steps in BEF‐A (left) and BEF‐N (right). (A–B) L7 transverse process (TP) after debridement. (C–D) Nerve retractor palpating the caudal L7 TP. (E–F) Identification of the L7 neuroforamen. (G–H) ClearCut Round Burr (4 mm, Arthrex) on the L7 TP base. (I–J) Final view of the L7 nerve root. Cranial is left, dorsal is up in all images.

**FIGURE 13:**
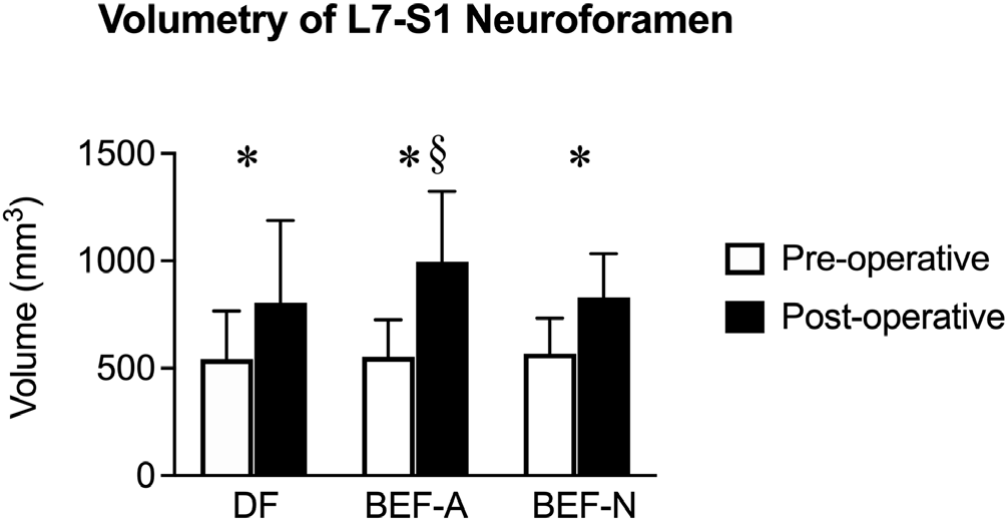
Bar graphs (mean ± SD) showing changes in neuroforaminal volume (mm^3^) for dorsolateral foraminotomy (DF), biportal endoscopic foraminotomy with a 3.0 mm arthroscope (BEF‐A), and biportal endoscopic foraminotomy with a nanoneedle scope (BEF‐N). * Significant volume increase after surgery; § Significantly more enlargement with BEF‐A than DF and BEF‐N.

**FIGURE 14:**
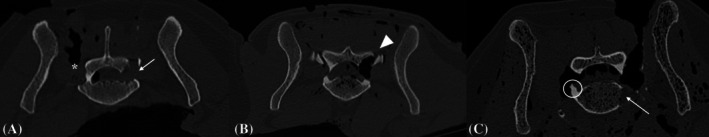
Examples of qualitative assessment of bone removal. (A) Appropriate bone removal in the cranial and ventral regions (white arrow) and insufficient bone removal in the cranial region (L7 pedicle; *). (B) Excessive bone removal in the dorsal region (partial L7–S1 facetectomy; white arrowhead). (C) Insufficient bone removal (retention of cranioventral boundary (“cranioventral lip”); white circle) and excessive bone removal (partial corpectomy of L7; white arrow) in the ventral region.

**FIGURE 15:**
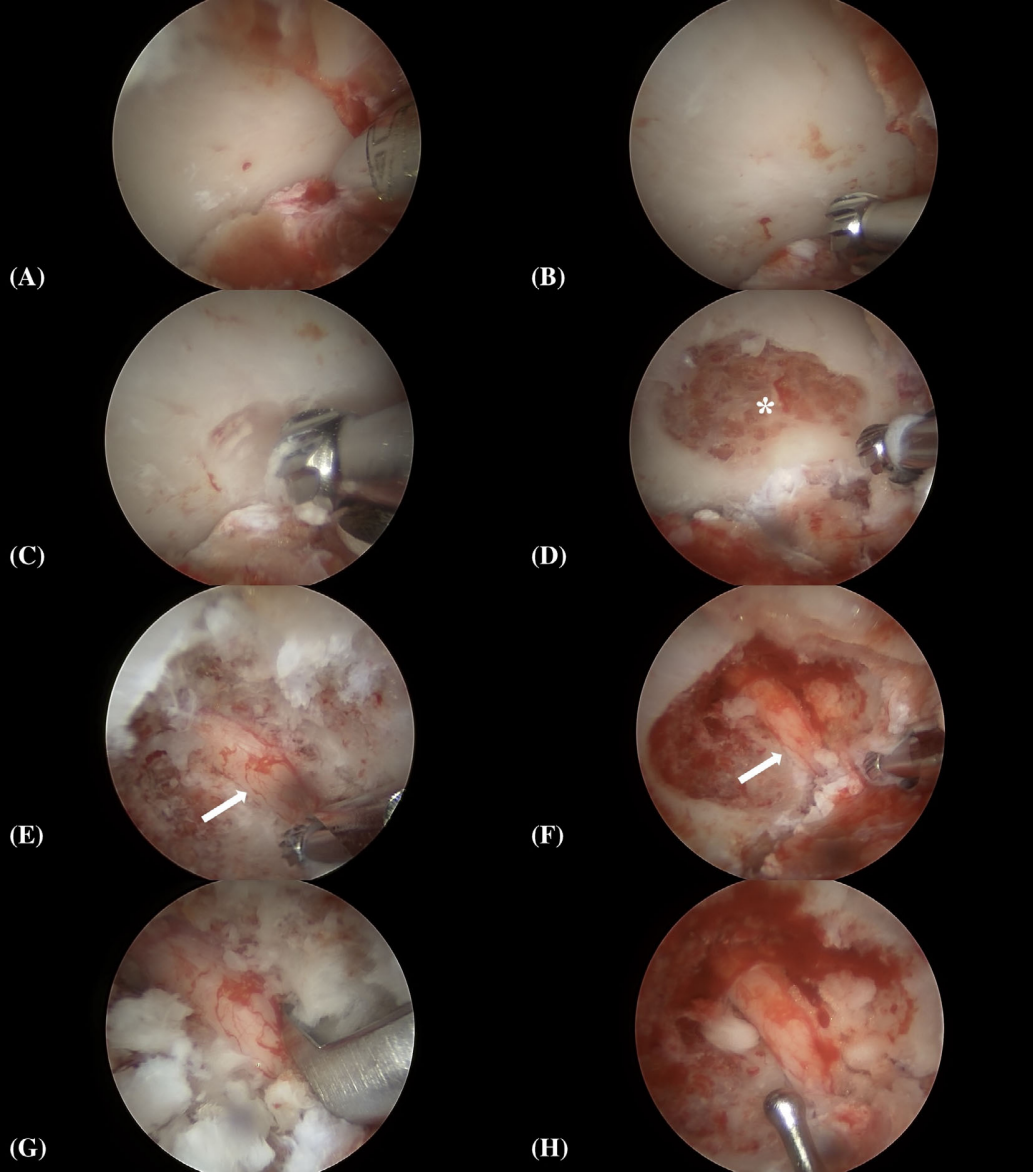
Intraoperative endoscopic views (BEF‐A) in a dog with L7–S1 foraminal stenosis: (A) L7 transverse process (TP) identified using a 3.5 mm Torpedo Shaver Tip (Arthrex). (B–C) 3.0 mm burr (Arthrex) placement and foraminotomy initiation. (D) Cancellous bone of the L7 pedicle (*). (E–F) L7 nerve root (arrow) and neuroforaminal exit zone. (G) Foraminotomy expansion using a 1 mm Kerrison rongeur. (H) Final view showing decompressed nerve root. Left is cranial and top is dorsal. Images were horizontally flipped for standardization.

**FIGURE 16:**
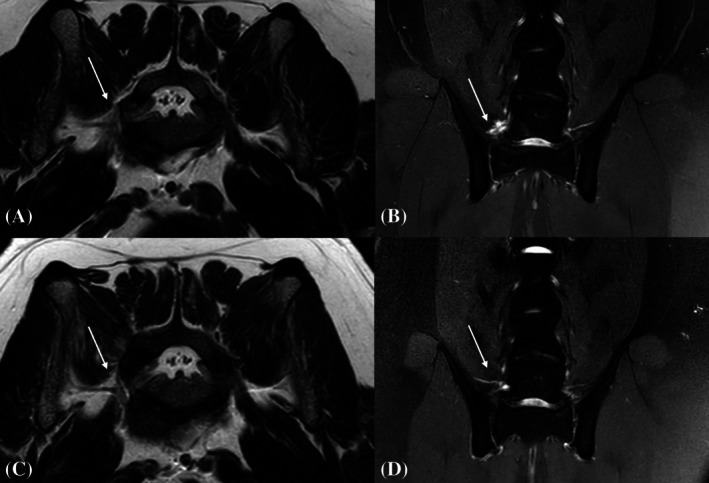
Magnetic resonance imaging (MRI) (T2 transverse; A, C) and T2‐SPIR dorsal (fat suppression sequence; B, D) of the L7–S1 neuroforamen of the patient treated by way of biportal endoscopic foraminotomy. Preoperative images (A, B) show moderate right‐sided neuroforaminal stenosis with enlargement of L7 nerve root with loss of surrounding fat signal and increased T2 signal intensity at the foraminal exit zone (arrows). Images attained 6 months after surgery (C, D) show a physiologic position of the L7 segmental nerve and absence of periforaminal signal abnormalities.

#### 3.2.5 Presence of iatrogenic L7 nerve root trauma

No macroscopic damage was detected for any of the procedures.

#### 3.2.6 CT‐based assessment

Volumetric assessment: All procedures resulted in significant enlargement of the neuroforamen (+59.7% ± 33.7%, *p* < .01; Figure 13). BEF‐A (+81.3% ± 30.0%) resulted in significantly greater enlargement compared to DF (+59.7% ± 33.7%, *p* = .04) and BEF‐N (+51.1% ± 38.8%, *p* = .04). No significant differences were observed between BEF‐N and DF (*p* = .86).

Qualitative assessment:

*Dorsal region*: for DF, bone removal in the dorsal region was classified as appropriate in 50.0% and excessive in 50.0%, involving removal of articular processes and/or dorsal lamina (Table 3, Figure 14). For BEF‐A, bone removal in the dorsal region was predominantly assessed as appropriate (87.5%), while in one case (12.5%) bone removal was graded as excessive (partial facetectomy). For BEF‐N, bone removal in the dorsal region was classified as appropriate in all cases (100%). No significant differences were found between the three procedures for qualitative evaluation of bone removal in the dorsal region (*p* > .21).

**TABLE 3:** Qualitative postoperative assessment of bone removal for dorsolateral foraminotomy (DF), biportal endoscopic foraminotomy with a 3.0 mm arthroscope (BEF‐A), and biportal endoscopic foraminotomy using a Nanoneedle (BEF‐N).

	CT evaluation of bone removal			
	Dorsal	Ventral	Cranial	Caudal
DF				
Appropriate	50.0% (4/8)	12.5% (1/8)	50.0% (4/8)	100% (8/8)
Insufficient	0% (0/8)	75.0% (6/8)	50.0% (4/8)	0% (0/8)
Excessive	50.0% (4/8)	12.5% (1/8)	0% (0/8)	0% (0/8)
BEF‐A				
Appropriate	87.5% (7/8)	50.0% (4/8)	100% (8/8)	100% (8/8)
Insufficient	0% (0/8)	37.5% (3/8)	0% (0/8)	0% (0/8)
Excessive	12.5% (1/8)	12.5% (1/8)	0% (0/8)	0% (0/8)
BEF‐N				
Appropriate	100% (7/7)	14.3% (1/7)	57.1% (4/7)	100% (7/7)
Insufficient	0% (0/7)	71.4% (5/7)	28.6% (2/7)	0% (0/7)
Excessive	0% (0/7)	14.3% (1/7)	14.3% (1/7)	0% (0/7)

*Note*: Bone removal was assessed as appropriate, insufficient, or excessive for the dorsal (facet joint and articular processes), ventral (cranioventral border of the neuroforamen, vertebral body of L7 and L7–S1 intervertebral disc), cranial (pedicle of L7), and caudal (pedicle of S1) regions of the L7–S1 neuroforamen.

Abbreviation: CT, computed tomography.

*Ventral region*: For DF, bone removal was most frequently classified as insufficient (75.0%), involving incomplete removal of the cranioventral border of the neuroforamen; excessive bone removal was observed in one specimen (12.5%), involving partial corpectomy of L7. For BEF‐A, bone removal was graded as appropriate (50.0%), insufficient (37.5%; incomplete removal of cranioventral border), and excessive (12.5%; partial corpectomy). For BEF‐N, most cases were categorized as insufficient (71.4%; incomplete removal of cranioventral border). No significant differences were found between the three procedures for the ventral region (*p* > .68).

*Cranial region*: For DF, bone removal was assessed as appropriate (50.0%) or insufficient (50.0%). For BEF‐A, all cases were classified as appropriate (100%). For BEF‐N, bone removal in the cranial region was assessed as appropriate (57.1%), insufficient (28.6%), and excessive (14.3%). No significant differences were found between the three procedures for bone removal in the cranial region (*p* > .18).

For all procedures, no bone removal was observed in the caudal region of the foramen (pedicle of S1) and was therefore classified as appropriate in all cases.

Subjectively, the greater increase in foraminal volume found for BEF‐A compared to DF could be attributed to more appropriate bone removal in the cranial (*p* = .18) and dorsal regions of the neuroforamen (*p* = .28).

### 3.3 Phase III: Surgical results for BEF‐A – clinical case report

The dog was anesthetized using routine procedures (Supplementary File S2). The lumbosacral region was clipped and aseptically prepared, followed by sterile draping. The dog was positioned in sternal recumbency with the hind limbs in a neutral position, the lumbosacral junction fixed in flexion, and the hindlimbs positioned alongside the trunk (Figure S1). Right‐sided BEF‐A using a 3.0 mm, 138 mm‐long, 30° oblique arthroscope (Arthrex) was performed using the methodology described above (Figure 15A–H).

Access to the L7 transverse process and neuroforamen was achieved without intraoperative complications. Debridement of the periforaminal soft tissues resulted in mild hemorrhage, leading to transient obstruction of endoscopic visualization. Hemorrhage was controlled using a bovine collagen‐based topical hemostatic agent (Lyostypt, B. Braun Melsungen AG, Melsungen, Germany) solubilized with cold (T = 4°C) sterile saline. The solubilized hemostatic agent was applied directly to the bleeding site through the keyhole port under arthroscopic guidance using a blunt‐tip cannula and left in place for approximately 2 min.

Foraminotomy was initiated as described, using 3.0 and 4.0 mm round arthroscopic burrs (Arthrex), which caused mild intraoperative osseous bleeding. Continuous endoscopic lavage prevented obstruction of the view during the burring procedure. BEF‐A provided good visualization of anatomical landmarks, the L7 nerve root, and the exit and middle zones of the foramen. Upon completion of the procedure, the L7 nerve root was free from exit zone compression or adhesions and could be freely mobilized outside the foramen (Figure 15H).

Closure was performed as described in Phase I, resulting in two surgical wounds measuring 7 mm and 9 mm for the cranial and caudal incisions, respectively.

#### 3.3.1 Postoperative course

The dog recovered uneventfully following surgery. Vital parameters remained within normal limits, and pain was effectively managed during the first 24 h with methadone (0.2 mg/kg IV every 4 h), robenacoxib (2 mg/kg orally once daily), and gabapentin (10 mg/kg orally three times daily). Six hours postoperatively, the dog was able to walk unsupported without signs of discomfort or lameness (Supplementary Video S5). One day postoperatively, the dog was fully ambulatory without signs of lameness or lumbosacral pain and was discharged from the hospital. Postoperative analgesia included robenacoxib (1 mg/kg orally once daily for 4 days) and gabapentin (10 mg/kg orally three times daily for 14 days). Postoperative management included strict rest with controlled leash walks (20 min, 3 times daily) for a period of 2 weeks.

#### 3.3.2 Follow‐up

At the 2‐week recheck examination, the dog remained free of lameness and lumbosacral pain. Physical examination was unremarkable, and the surgical wounds had healed without complications. The owner reported complete resolution of the prior signs of discomfort and a marked improvement in mobility. The postoperative DLSS score was 19/21. A gradual return to normal activity over the following 4 weeks was recommended, and analgesic medications were discontinued.

At the 6‐month follow‐up, the dog had resumed full functional activity with no recurrence of clinical signs. The clinical DLSS score was 21/21. MRI of the lumbosacral junction revealed a morphologically normal right‐sided L7 segmental nerve without T2‐signal hyperintensity (Figure 16).

## 4 DISCUSSION

In Phase I, a minimally invasive technique for BEF using arthroscopic equipment was developed, supporting our first hypothesis. In Phase II, BEF‐A and BEF‐N enabled better direct visualization of the L7 nerve root and foraminal zones without risking iatrogenic injury. BEF‐A also resulted in greater neuroforaminal enlargement than DF, confirming our second hypothesis. In Phase III, BEF‐A was successfully used to treat a dog with L7–S1 foraminal stenosis, thus supporting our third hypothesis.

### 4.1 Establishment of minimally invasive BEF

The rationale of the present work was to provide a procedure that can be performed using arthroscopic equipment, that is readily available in most veterinary hospitals, rather than dedicated endoscopic spinal instrumentation. By intentionally focusing on a surgical technique using arthroscopy equipment, the aim was to maximize clinical applicability and accessibility. Recently, a biportal endoscopic technique for foraminotomy of L7–S1 was published, using specialized spinal endoscopic equipment including a 4.0 mm, 0° spinal endoscope and serial portal dilators.^22^ The current study aimed to describe a specialized surgical technique with arthroscopic equipment, using a 30° arthroscope and a 0° Nanoneedle scope and the specific surgical steps associated with this procedure. The importance of accurate and detailed description of arthroscopic surgical techniques for spinal surgery is also reflected in the human literature, where several minimally invasive spinal surgery techniques using arthroscopic equipment have recently been published.^28^

Both the 3.0 mm 30° arthroscope and NanoNeedle Scope proved feasible for safe and effective L7 nerve root decompression. Challenges and complications encountered in Phase I—such as L7 transverse process fracture and starting the procedure at the incorrect location—highlight the importance of precise localization, clear anatomical visualization, and the need for thorough preoperative three‐dimensional (3D) planning, intraoperative imaging, and endoscopic expertise. Thus, intraoperative fluoroscopy and intermittent probing of the neuroforamen with a nerve hook were key components of this procedure.

Although the absence of a joint space differentiates BEF from arthroscopy, the surrounding muscle and vertebrae allowed the establishment of a confined environment conducive to applying arthroscopic principles. Irrigation was necessary to create a 3D working space, which allowed for the identification of key anatomical structures and improved visibility in an underwater environment. However, the use of continuous irrigation may result in local compression at the surgical site and increased intraspinal pressure cranial to the lumbosacral region. During the procedure, continuous outflow was observed at the portal sites, indicating effective fluid egress from the working space around the nerve root and neuroforamen. Moreover, human studies evaluating biportal and interlaminar endoscopic spinal procedures have demonstrated that low or conservative irrigation pressures (<30 mmHg) are associated with smaller increase in epidural and intracranial pressures and a significantly reduced risk of pressure‐related complications.^26^, ^27^ Based on these findings, an irrigation pressure of 15 mmHg was selected, representing a conservative setting well below commonly reported thresholds while still allowing adequate visualization and effective debris removal. The irrigation flow rate was set at 25 mL/min and adjusted progressively intraoperatively to achieve optimal visualization while maintaining good outflow. At no point was impaired drainage or excessive tissue distension observed. Given the low irrigation pressure, controlled flow rate, and preserved outflow the risk of fluid‐induced compression of the spinal nerve roots and spinal cord was considered minimal.

### 4.2 Surgical time, invasiveness, and visualization of the L7 nerve root and neuroforaminal zones

All three techniques effectively enlarged the L7–S1 neuroforamen. However, BEF and DF differed in surgical time, visualization, and invasiveness.

DF was faster, owing to the surgeon's familiarity with the procedure. BEF‐A and BEF‐N took longer due to the novelty and learning curve of minimally invasive spinal surgery. BEF‐N was deemed more challenging due to its lower image resolution and narrower field of view with a 0° arthroscope, impairing spatial orientation. In human neurosurgery, proficiency in endoscopic foraminotomy improves after ~20 cases, with surgical times approaching those of open techniques.^8^, ^9^, ^10^, ^11^ A similar trend was observed here, as operative times halved over the course of the study, and the learning curve of minimally invasive spinal surgery may be steep as reported for other MI techniques.^29^

BEF incisions were significantly smaller than DF incisions (<1.0 cm), with no need for extensive soft tissue retraction. These features may reduce tissue trauma, pain, hospitalization time, and enable outpatient treatment.

BEF‐A and BEF‐N offered superior nerve root and foraminal visualization. Unlike DF, which relies on palpation and nerve retraction, BEF provided continuous visual access to the middle and exit zones, improving precision and reducing nerve manipulation.

### 4.3 Efficacy for neuroforaminal enlargement

All techniques resulted in significant enlargement of the neuroforamen. BEF‐A produced the most substantial expansion, likely owing to superior visualization afforded by telescopic magnification, the clarity of a fluid‐cleared underwater arthroscopic image, and the enlarged field of view provided by the 30° angled arthroscope. These advantages enabled precise, targeted removal of the exit and middle zone boundaries. In contrast, the entry zone of the foramen was poorly visualized across all procedures, consistent with the limitations inherent to the outside‐in approach. However, DF and BEF‐A are mainly directed at decompressing the exit and middle zones of the foramen, aligning with the current understanding that L7–S1 foraminal stenosis in dogs typically affects the foraminal exit zone.^2^

Care should be taken with respect to excessive and insufficient bone removal while performing foraminotomy at L7–S1. Although all procedures were effective in enlarging the volume of the neuroforamen, qualitative assessment of bone removal revealed insufficient or excessive bone resection in some cases. Excessive or insufficient bone removal in the dorsal, ventral and cranial regions of the neuroforamen were surgical errors, which were generally not noticed during the surgical procedure, but instead mainly observed on postoperative CT imaging. With respect to insufficient bone removal, the cranioventral boundary of the neuroforamen was most frequently not sufficiently removed (75.0%, 37.5%, and 71.4% for DF, BEF‐A and BEF‐N, respectively). A possible explanation is that the predominantly craniodorsal trajectory used to follow the course of the neuroforamen may leave the cranioventral boundary inadequately removed. Removal of the cranioventral boundary (“cranioventral lip”) of the foramen is crucial for achieving sufficient decompression in clinical cases.

Excessive bone removal was most often observed in the dorsal region (50%, 12.5% and 0% for DF, BEF‐A and BEF‐N, respectively) followed by the ventral region (12.5%, 12.5% and 14.7% for DF, BEF‐A, and BEF‐N, respectively). Excessive removal of essential stabilizing structures in these regions, such as the facet joints, may induce post‐operative instability of the lumbosacral junction, especially in bilateral procedures. Excessive bone removal in the cranial region was only observed in one specimen (BEF‐N). Excessive bone removal in the cranial region, involving partial pediculectomy, should also be avoided with respect to future revision surgery involving pedicle screw fixation of the lumbosacral junction.

Excessive and insufficient bone removal were largely detected on post‐operative imaging and not during the actual surgical procedure. For the above‐described reasons, postoperative CT evaluation of foraminotomy may be beneficial in clinical cases to fully assess the extent of appropriate and excessive/insufficient foraminal enlargement.

### 4.4 Biportal versus monoportal endoscopic spine surgery

In the field of minimally invasive spinal surgery, a distinction between uniportal and biportal endoscopic spine surgery can be made.^30^, ^31^, ^32^ Monoportal endoscopic procedures employ a single working channel and have been associated with reduced postoperative pain and limited soft‐tissue disruption; however, they require specialized endoscopic systems and advanced technical expertise, resulting in a relatively steep learning curve.^30^, ^31^, ^32^ Biportal endoscopic spine surgery utilizes separate portals for visualization and instrumentation, allowing improved instrument maneuverability and the use of conventional surgical tools.^31^, ^32^ However, biportal approaches are associated with at least two access ports, which may increase approach‐related iatrogenic tissue trauma when compared with monoportal techniques.^31^ Despite these differences, comparative studies in human spinal surgery report largely comparable clinical outcomes between monoportal and biportal approaches.^30^, ^31^, ^32^ The technique described in the present study follows a biportal concept and can be performed using relatively simple equipment that is readily available in most veterinary hospitals. As minimally invasive spinal surgery continues to evolve in small animal surgery, it is expected that more specialized equipment, including monoportal endoscopic systems, will become increasingly available to the veterinary practitioner.

### 4.5 Clinical application of BEF‐A

BEF‐A was successfully used to treat a dog with degenerative L7–S1 foraminal stenosis. BEF‐A was preferred over BEF‐N due to its better image quality and field of view and higher efficacy as evidenced in Phase II. The procedure was performed without major complications and allowed excellent visualization of the nerve root and foramen. Hemorrhage during soft tissue debridement and pedicle burring was controlled using soluble bovine collagen through the keyhole port. In human endoscopic surgery, dedicated hemostasis techniques such as radiofrequency ablation are effectively applied to address such complications. Similar tools are under evaluation for use in BEF in dogs.

## 5 LIMITATIONS

This study had several limitations. First, all procedures were performed on healthy cadaveric spines. In cases of advanced DLSS, lumbosacral transitional vertebrae, or nerve root pathology, BEF may be more technically demanding. More specifically, the consequences of persistent inflammation—such as the development of fibrotic tissue and hypertrophy of soft‐tissue structures—may reduce the available working space, thereby complicate anatomical identification. In addition, probing of the foraminal exit zone and the foramen course may be significantly complicated by DLSS patients with significant radiculoneuritis and may increase the risk of iatrogenic nerve root injury. Moreover, in the present study, the dorsal branch of the nerve root was neither specifically visualized nor directly examined. Soft‐tissue preparation was focused on the exit zone of the neuroforamen, with deliberate efforts to minimize damage to adjacent structures. Nevertheless, the dorsal branch of the nerve root may have been at risk of iatrogenic injury during soft‐tissue debridement, although this structure was not identified in any of the ex vivo or in vivo procedures performed. Iatrogenic nerve root injury was assessed macroscopically. However, the gold standard for the assessment of acute and chronic iatrogenic nerve root damage is histological examination of the nerve root. Due to autolytic changes, lack of inflammatory reactions post mortem, and poor histologic quality in ex vivo specimens, histology was not performed. Future clinical studies assessing acute and chronic neuropathy post BEF are warranted. Additionally, intraoperative complications such as hemorrhage may pose greater challenges.

Nevertheless, BEF‐A was successfully applied in a clinical case. Further in vivo studies on a larger patient cohort are needed to evaluate efficacy, safety, and complications.

## 6 CONCLUSION

A surgical technique for BEF using arthroscopic equipment was successfully established. BEF‐A offers superior visualization and efficacy with significantly less soft tissue trauma compared to DF. It was successfully applied in a clinical case of L7–S1 foraminal stenosis and is proposed as a novel, minimally invasive alternative to conventional DF.

## AUTHOR CONTRIBUTIONS

Bekiaridis D, DVM: Responsible for study logistics, assisting in all surgical procedures, data collection and analysis, drafting the manuscript. Pozzi A, DVM, MS, DACVS (Small Animal), DECVS, DACVSMR, ACVS Founding Fellow MIS (Small Animal Orthopedics): Involved in study design, data interpretation, drafting the manuscript. Steffen F, DVM, DECVN: Involved in performing the surgical procedures data interpretation and drafting the manuscript. Guevar J, PhD, DECVN: Involved in study design, data interpretation, and drafting the manuscript. Smolders LA, DVM, PhD, DECVS: Responsible for funding acquisition and study supervision, involved in the design of the study, performing the surgical procedures, data collection and analysis, drafting and editing of the manuscript. All authors provided a critical review of the manuscript and endorse the final version. All authors are aware of their respective contributions and have confidence in the integrity of all contributors.

## FUNDING INFORMATION

The cadaveric part of this project received funding support from Arthrex GmbH (Study ID: IIRR‐01785). Professor Antonio Pozzi is a paid consultant for Arthrex Vet Systems.

## CONFLICT OF INTEREST STATEMENT

This study was supported by Arthrex Vet Systems who supplied the arthroscopic equipment (Nanoneedle, Shaver Tips) free of charge.

## Supporting information


**Figure S1.** Supplementary Table.

**File S1.** Surgical procedure.

**File S2.** Instrumentation list.

**File S3.** Anesthesia protocol.

**Table S1.** Specimen list.

**Video S1.** Soft tissue debridement.

**Video S2.** Foraminotomy with Burr.

**Video S3.** Foraminotomy with Kerrison.

**Video S4.** Probing of L7 nerve root.

**Video S5.** Case report postOP walking.

## Data Availability

Encourages data sharing.
