# Supplementary figures and images for: Biportal endoscopic foraminotomy of the L7–S1 neuroforamen in dogs: Description of surgical technique and ex vivo comparison with conventional open dorsolateral foraminotomy
Source: Vet Surg. 2026 Mar 12;55(4):837–55. doi: 10.1111/vsu.70096 (PMC13150048; doi:10.1111/vsu.70096)

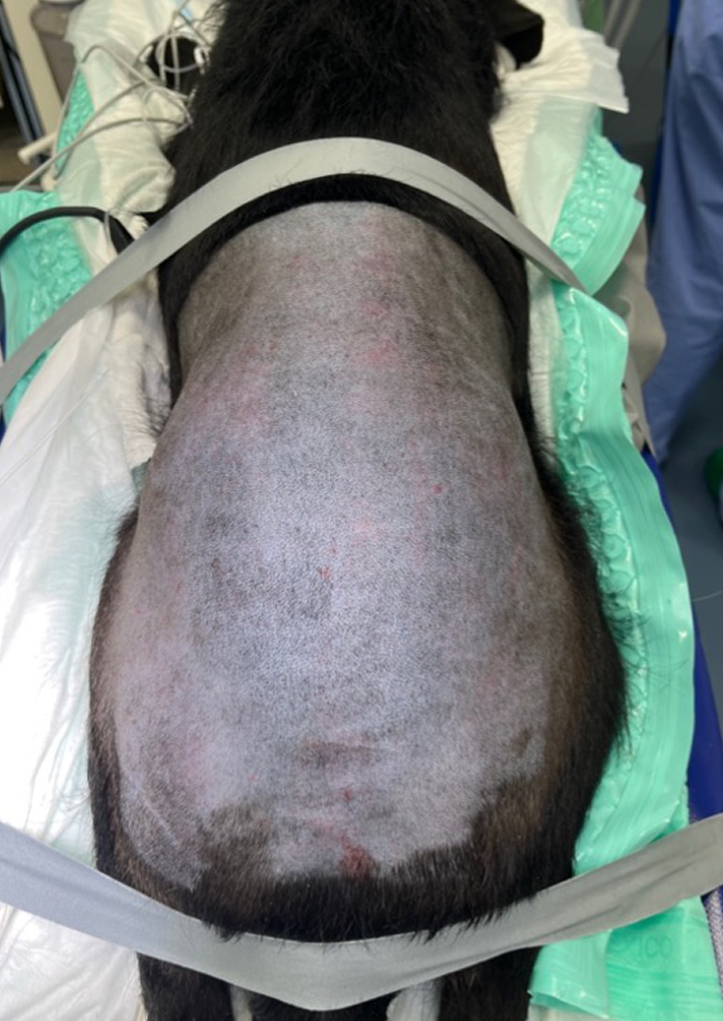

Supplement: Supplementary file 1 — Figure S1. Supplementary Table. [file VSU-55-837-s004.tif]

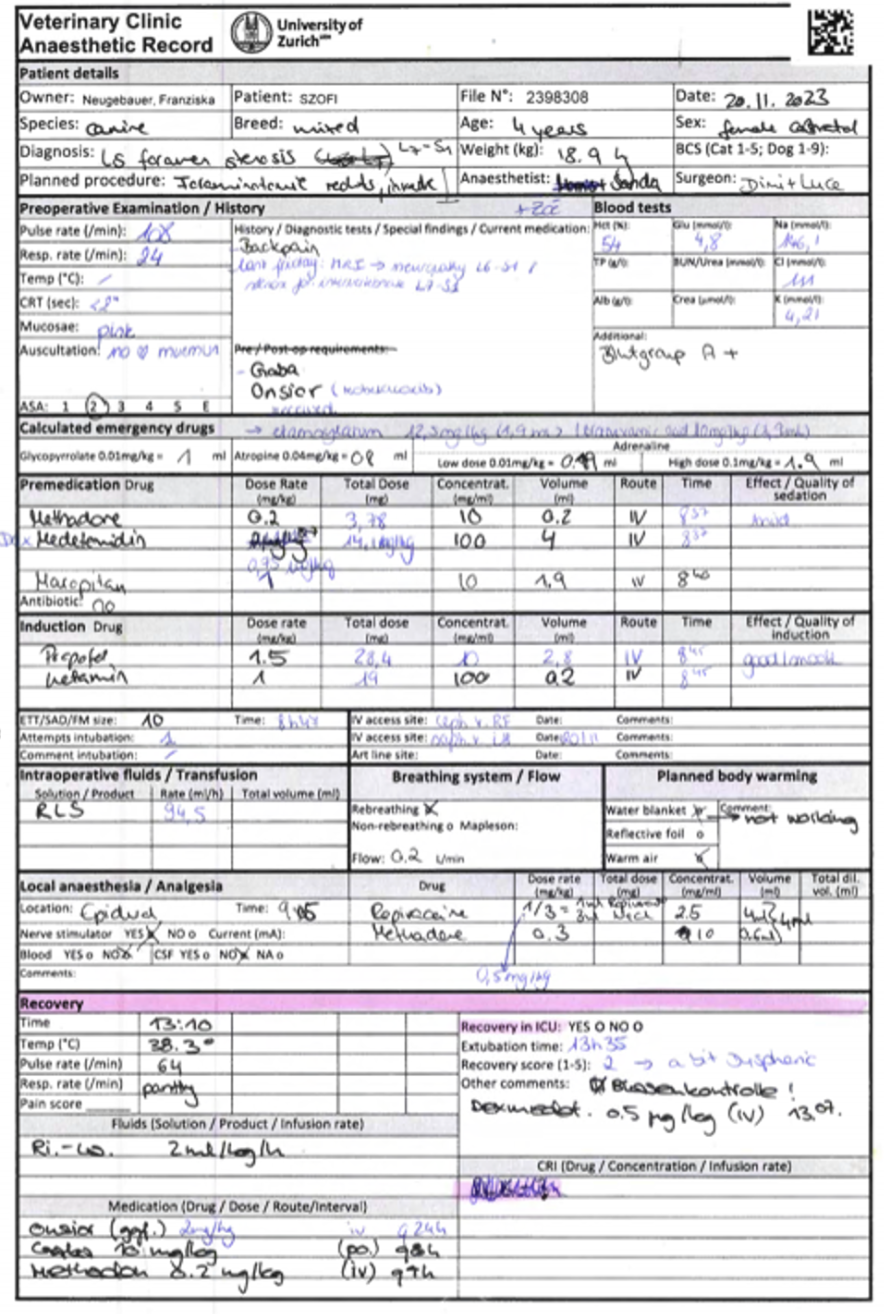


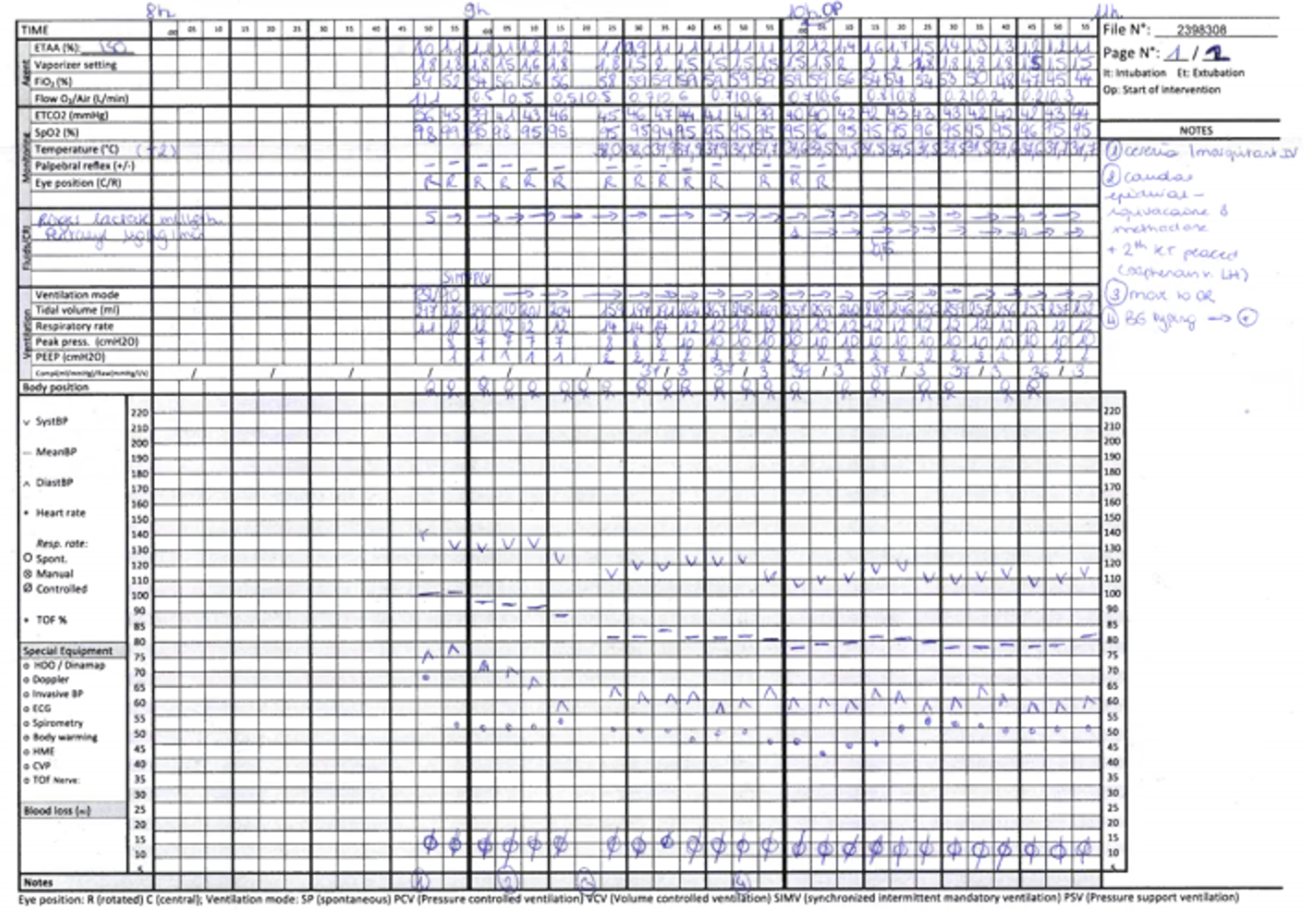


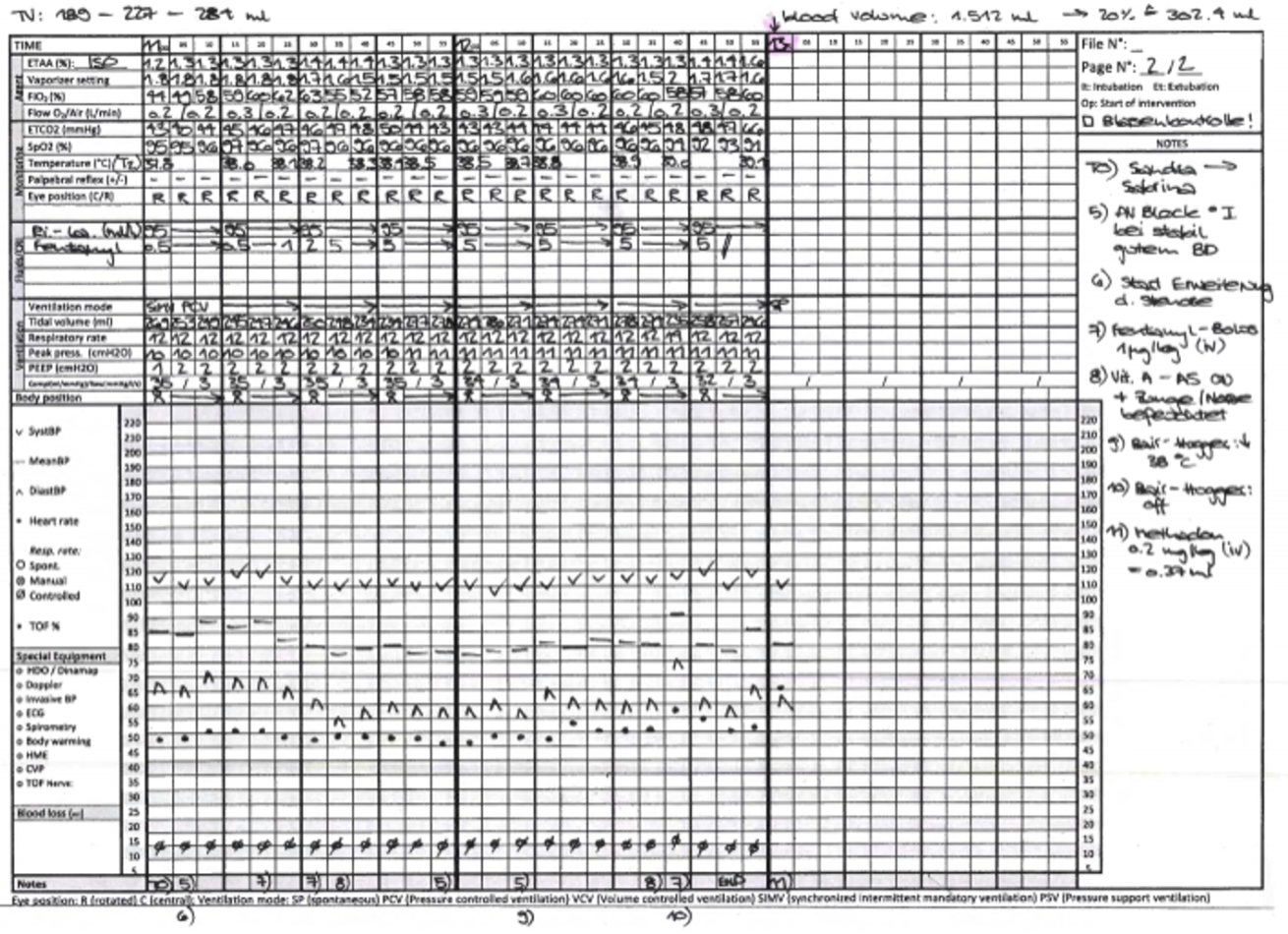

Supplement: Supplementary file 4 — File S3. Anesthesia protocol. [file VSU-55-837-s010.docx]

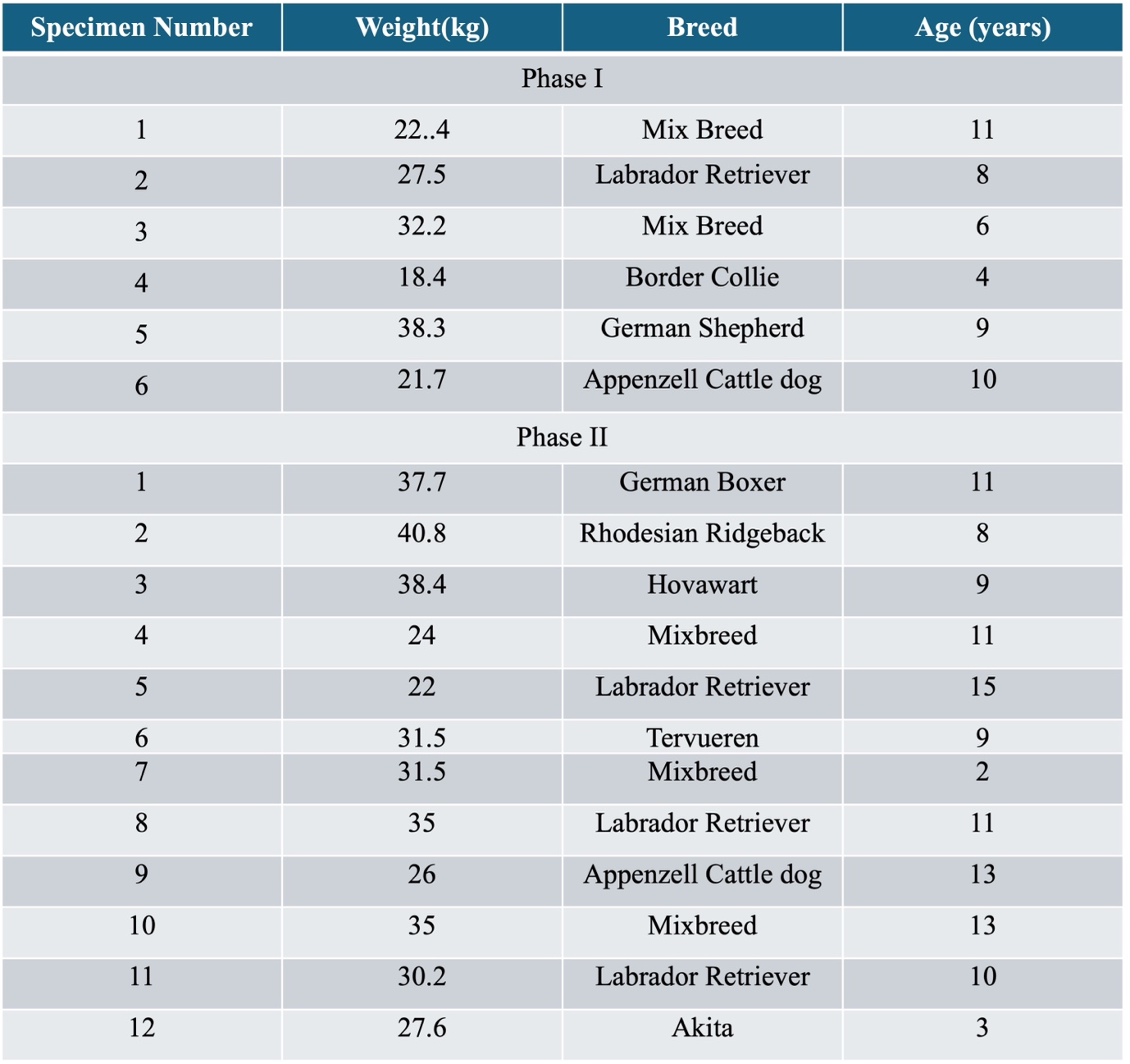


Supplementary Table 1. Specimen list

Supplement: Supplementary file 5 — Table S1. Specimen list. [file VSU-55-837-s008.docx]
