# Supplementary material for: Biportal endoscopic foraminotomy of the L7–S1 neuroforamen in dogs: Description of surgical technique and ex vivo comparison with conventional open dorsolateral foraminotomy
Source: Vet Surg. 2026 Mar 12;55(4):837–55. doi: 10.1111/vsu.70096 (PMC13150048; doi:10.1111/vsu.70096)
Supplement: Supplementary file 2 — File S1. Surgical procedure. [file VSU-55-837-s003.docx]

**Supplementary file 1: Surgical Procedure**

1. Patient positioning: The patient was positioned in sternal recumbency, allowing identification of the surgical landmarks (Figure 2A). Sandbags were placed laterally to the patient to prevent spinal rotation. The lumbosacral joint was positioned in flexion in order to increase the working space surrounding the L7 nerve root.
2. Position of the surgeon: The surgeon was standing lateral to the patient on the side requiring foraminotomy (Figure 2B).

The definitive procedure for BEF involved the following steps (for a detailed list of surgical equipment: Supplementary File 1):

- Step 1. The following anatomical landmarks were identified and marked using a surgical skin marker (Figure 2A):

-Craniodorsal aspect of the iliac wing

-Dorsal spinous process of L6, L7 and S1 vertebrae

- Step 2: The base of the L7 transverse process was identified using a hypodermic needle (20G × 2¾" (0.9 mm × 70 mm). The needle was inserted until a clear bony resistance was felt. Fluoroscopic control of correct localization was performed (Figure 3A). Similarly, the position of the cranial edge of the sacro-iliac joint was identified and fluoroscopically confirmed.
- Step 3: Establishment of keyhole port:
  A stab incision of approximately 1 cm (craniocaudal direction) was made through the skin, subcutis and lumbosacral fascia, directed parallel to the hypodermic needle inserted in the base of the L7 transverse process using a No.11 scalpel blade (Figure 3B). Straight hemostatic forceps were inserted through the incision, pushing the tips of the forceps onto the bone of the base of the L7 transverse process. The hemostatic forceps was opened multiple times in various directions to bluntly remove soft tissue covering the base of the L7 transverse process. The hemostatic forceps was removed, and the arthroscope was inserted through the incision (Figure 3C). Sterile ringer’s lactate solution (Fresenius Kabi, Lake Zurich, IL, USA) was continuously infused through the arthroscopic sheath with the DualWave Arthroscopy Fluid Management System (Arthrex) at a pressure of 15 mmHg to expand the endoscopic working space and to enhance surgical visualization. Continuous egress of the fluid from the surgical field was maintained through the keyhole port, which, due to its width, allowed continued egress of excessive fluid.

The establishment of the dorsal keyhole port was considered completed when the basis of the L7 transverse process was visually confirmed using the endoscope (Figure 3D).

- Step 4: Establishment of the caudal port:
  Similar to the keyhole port, the cranial border of the sacro-iliac joint was identified using a hypodermic needle under fluoroscopic control and a stab incision was performed (Figure 4A). A 2.9 mm Switching Stick was placed through the incision in a 45° angle to the spine, in a cranioventral direction, aiming at the base of the L7 transverse process (Figure 4B). The placement of the tip of the switching stick on the transverse process was visually confirmed using the arthroscope (Figure 4C). Subsequently, the arthroscope was removed from the keyhole incision and replaced into the caudal incision using the switching stick as a guide (Figure 4D-4E). The base of the transverse process could now be visually confirmed from the caudal port (Figure 4F). The arthroscope remained in the caudal port for the rest of the procedure, unless a different angle of visualization of the neuroforamen was required.
- Step 5: Soft tissue debridement: The arthroscopic power shaver handpiece (APS II Shaver Handpiece, Arthrex) was inserted through the dorsal keyhole port (Figure 5A). Debridement of the peri-foraminal soft tissue structures (Figure 5B) surrounding the caudal aspect of the basis of the L7 transverse process was performed under continuous suction using a Torpedo Blade Shaver Tip 3.5 mm (Arthrex) (Figure 5C; Supplementary Video 1), until the boundaries of the neuroforamen could be clearly identified (Figure 5D).
- Step 6: Confirmation of the location of the L7-S1 neuroforamen was performed by carefully inserting a nerve hook retractor into the L7-S1 neuroforamen in a craniodorsal direction, aiming away from the course of the L7 nerve root (Figure 6A). Correct localization was confirmed by performing a single fluoroscopy radiograph (Figure 6B).
- Step 7: Initiation of the foraminotomy at the level of the caudal aspect of the L7 transverse process in a craniodorsal direction, into the exit zone of the neuroforamen using a Clear-Cut Round Burr Shaver Tip (Size: 4 mm, Arthrex Vet Systems, Naples, FL) attached to the Shaver Handpiece, inserted through the dorsal keyhole incision (Figure 7A-D). The direction of burring was guided by identifying the direction of the neuroforamen by way of intermittent probing it with the nerve hook. The transparent protective sheath of the shaver tip offered additional protection of the L7 nerve root as the foraminotomy progressed cranially (Supplementary Video 2).
- Step 8: Once the boundaries of the foraminotomy had been created and the L7 nerve root could be clearly visualized, the foraminotomy was expanded cranially and medially using a smaller sized, 3 mm arthroscopic Burr and Kerrison rongeurs 1 mm (Figure 8A-D); Supplementary Video 3).
- Step 9: Determination of endpoints (Figure 9A-C). The procedure was considered complete when:
- The nerve root was clearly free of any cranial, ventral and medial impingements and freely movable after gentle traction using the nerve hook retractor.
- The dorsal root ganglion was identified and could be moved freely out of the neuroforamen (Supplementary Video 4).
- Step 10: Closure was performed in routine fashion by suturing the lumbosacral fascia (Monoplus 3-0, B. Braun Surgical S.A., Rubi, Spain), the subcutis (Monosyn 3-0, B. Braun) and the skin (Supramid 3-0, B. Braun) with single interrupted sutures.
