# Supplementary material for: Biportal endoscopic foraminotomy of the L7–S1 neuroforamen in dogs: Description of surgical technique and ex vivo comparison with conventional open dorsolateral foraminotomy
Source: Vet Surg. 2026 Mar 12;55(4):837–55. doi: 10.1111/vsu.70096 (PMC13150048; doi:10.1111/vsu.70096)
Supplement: Supplementary file 3 — File S2. Instrumentation list. [file VSU-55-837-s006.docx]

**Supplementary File 2**

**Instrumentation List**

The following arthroscopes and instrumentation for BEF were selected:

1. Arthroscopes
   1. Arthroscope, 30°, 3mm X 138mm
      1. Arthrex Monitor, 4K, 32¨
      2. Synergy^UHD4^ Imaging Platform
      3. Fused light Guide, Wolf 3.5mm X 274cm
      4. Sheath, High-Flow, 2 Stopcock, for 3mm Arthroscope
      5. Conical Obturator, for 3mm Scope Sheath w/Handle
      6. Switching stick, 2.9mm X 305mm
   2. NanoNeedle Scope, 0°, 2.0mm X 125mm with NanoScope Tablet Control Unit
2. Water Pump, DualWave Arthroscopy Fluid Management System
3. Arthroscopic Shaver and Shaving Tips
   1. Synergy Resection Shaver Console
   2. APS II Shaver Handpiece
   3. APS II Footswitch Control Standard
   4. Burr, Round 10 Flute 3.0mm
   5. Burr, ClearCut Round, 8 Flute, 4.0mm X 13cm
   6. Torpedo, 3.5mm X 13cm
4. Suction Device, connected with suction cable to the handpiece. (Medap Twista SP 1070, Atmos)
5. Laminectomy Instrumentation
   1. Kerrison Rongeurs 1mm (normal and reverse tip)
   2. Long and blunt right angled nerve root retractor
   3. Adson and Freer periosteal elevators
   4. Straight Hemostatic forceps (Mosquito)
   5. Scalpel blade holder and scalpel blade No11
   6. Hypodermic needle (20G × 2¾" (0.9 mm × 70 mm)
6. Fluoroscopy/C-Arm (Veradius Neo 718 131 Mobile C-Arm, Philips)
